# SF3B3-regulated mTOR alternative splicing promotes colorectal cancer progression and metastasis

**DOI:** 10.1186/s13046-024-03053-4

**Published:** 2024-04-26

**Authors:** Tong Xu, Xichuan Li, Wennan Zhao, Xue Wang, Leixin Jin, Zhiqiang Feng, Huixiang Li, Mingzhe Zhang, Yiqing Tian, Ge Hu, Yuan Yue, Xintong Dai, Changliang Shan, Weihua Zhang, Chunze Zhang, Youcai Zhang

**Affiliations:** 1 School of Pharmaceutical Science and Technology, Tianjin University, Tianjin, 300072 China; 2 Tianjin Key Laboratory of Animal and Plant Resistance, College of Life Sciences, Tianjin Normal University, Tianjin, 300382 China; 3 Cancer Biology Program, University of Hawaii Cancer Center, Honolulu, HI 96813 USA; 4 Department of Colorectal Surgery, Tianjin Union Medical Center, Tianjin, 30021 China; 5 State Key Laboratory of Medicinal Chemical Biology, College of Pharmacy and Tianjin Key Laboratory of Molecular Drug Research, Nankai University, Tianjin, 300350 China; 6 Tianjin Haihe Hospital, Tianjin, 300051 China

**Keywords:** SF3B3, mTOR, Colorectal cancer, Metastasis, Alternative splicing

## Abstract

**Background:**

Aberrant alternative splicing (AS) is a pervasive event during colorectal cancer (CRC) development. SF3B3 is a splicing factor component of U2 small nuclear ribonucleoproteins which are crucial for early stages of spliceosome assembly. The role of SF3B3 in CRC remains unknown.

**Methods:**

SF3B3 expression in human CRCs was analyzed using publicly available CRC datasets, immunohistochemistry, qRT-PCR, and western blot. RNA-seq, RNA immunoprecipitation, and lipidomics were performed in *SF3B3* knockdown or overexpressing CRC cell lines. CRC cell xenografts, patient-derived xenografts, patient-derived organoids, and orthotopic metastasis mouse models were utilized to determine the in vivo role of SF3B3 in CRC progression and metastasis.

**Results:**

SF3B3 was upregulated in CRC samples and associated with poor survival. Inhibition of SF3B3 by RNA silencing suppressed the proliferation and metastasis of CRC cells in vitro and in vivo, characterized by mitochondria injury, increased reactive oxygen species (ROS), and apoptosis. Mechanistically, silencing of *SF3B3* increased mTOR exon-skipped splicing, leading to the suppression of lipogenesis via mTOR-SREBF1-FASN signaling. The combination of *SF3B3* shRNAs and mTOR inhibitors showed synergistic antitumor activity in patient-derived CRC organoids and xenografts. Importantly, we identified SF3B3 as a critical regulator of *mTOR* splicing and autophagy in multiple cancers.

**Conclusions:**

Our findings revealed that SF3B3 promoted CRC progression and metastasis by regulating mTOR alternative splicing and SREBF1-FASN-mediated lipogenesis, providing strong evidence to support SF3B3 as a druggable target for CRC therapy.

**Supplementary Information:**

The online version contains supplementary material available at 10.1186/s13046-024-03053-4.

## Background

Colorectal cancer (CRC) ranks the second most commonly diagnosed cancer and the third-highest cause of cancer-related deaths worldwide [1]. Remarkably, at the time of diagnosis, 10–30% of CRC patients present with distant metastasis, with approximately 50% developing liver metastasis, the leading cause of mortality in CRC patients [2]. While surgical resection is considered the sole potentially curative therapy for CRC, many advanced CRC patients require chemotherapy. Nevertheless, only a small percentage of CRC patients with metastasis manage to survive for more than 5 years even after chemotherapy or surgery. The challenge in achieving effective CRC therapy stems largely from a limited understanding of the fundamental pathological mechanisms underlying CRC tumorigenesis and metastasis.

More than 90% of mammalian genes undergo alternative splicing (AS), a process through which several mRNA variants can be generated from a single gene. AS is executed by a sophisticated spliceosome machinery, which recognizes different splice sites and catalyzes two fundamental transesterification reactions. The core spliceosome comprises five small nuclear ribonucleoprotein (snRNPs) complexes (U1, U2, U4, U5, and U6 snRNP) and over 200 related proteins. Alterations in either cis-acting RNA sequence elements or trans-acting regulation splicing factors result in splicing dysregulation, which underlies various human diseases, including cancer [3]. Accumulating evidence suggests that aberrant AS is associated with CRC proliferation, invasion, apoptosis, angiogenesis, and drug-resistance [4–6].

The mammalian or mechanistic target of rapamycin (mTOR) is a serine/threonine kinase involved in regulating autophagy, metabolism, survival, and the immune response. Hyperactivation of the mTOR pathway contributes to tumor initiation and progression [7, 8]. Targeting mTOR has emerged as an effective therapeutic approach for various cancers, including CRC [9]. While several mTOR splicing variants are documented in database, only two functional human mTOR isoforms have been identified, namely mTORα and mTORβ [10]. To date, the splicing mechanism for mTORβ as well as the role of mTOR splicing in tumor development remain elusive.

The SF3b complex is an intrinsic component of the U2 snRNP and plays a crucial role in recognizing the branch point sequence (BPS) during early stages of spliceosome assembly [11]. The SF3b complex comprises seven proteins (SF3B1, SF3B2, SF3B3, SF3B4, SF3B5, SF3B6, and SF3B7), each demonstrated to possess distinct structures and functions. SF3B1 and SF3B7 (also known as PHD finger protein 5A, PHF5A) have been extensively investigated for their functional roles in multiple cancers, including CRC [4, 12–15]. However, whether other SF3b components play a role in CRC remains unknown. In this study, we systematically investigated the role of SF3B3 in regulating AS events and gene expression in CRC cells. We reported that *SF3B3* overexpression was common in CRC patients, and was clinically related with CRC prognosis. SF3B3 facilitated the proliferation and metastasis of CRC cells in vitro and in vivo. Mechanistically, SF3B3 promoted lipogenesis by regulating mTOR-SREBF1-FASN signaling. Importantly, we identified SF3B3 as a critical regulator of mTOR splicing and autophagy in multiple cancers, including CRC.

## Materials and methods

### Animal studies

BALB/C nude mice (male and female, 4–5 weeks of age) were obtained from Beijing Vital River Laboratory Animal Technology Co., Ltd. (Beijing, China). NSG mice (female, 6–8 weeks of age) were obtained from Gem Pharmatech (Jiangsu, China). The protocol for mouse housing and usage was approved by the Laboratory Animal Ethics Committee of Tianjin Haihe hospital (Tianjin, China).

### Cell culture

The human CRC cell lines LoVo, HT29, SW480 and other cancer cell lines were obtained from National Infrastructure of Cell Line Resource (Beijing, China). The HEK293T cell line was acquired from Cell Resource Center of Shanghai Institutes for Biological Sciences (Shanghai, China). LoVo, SW480, Hela, MCF7, Huh7, and HEK293T cells were cultured in Dulbecco’s modified Eagle’s medium (DMEM) supplemented with 10% fetal bovine serum (Cell-Box, AUS-01S-02) and 1% penicillin/streptomycin. HT29 cells were maintained in RPMI-1640 medium supplemented with 10% fetal bovine serum and 1% penicillin/streptomycin. All cells were incubated in a humidified incubator at 37 °C with 5% CO_2_.

### Human samples and ethical statement

A total of 127 paired human CRC and adjacent noncancerous tissues were obtained from Tianjin Union Medical Center (Tianjin, China). Among these, 98 paired human CRC and adjacent noncancerous tissues were utilized for tissue microarray, while 25 paired tissues were used for qRT-PCR and western blot analysis. Three patient CRC tissues obtained at the time of surgery were used for constructing PDX models. One patient CRC tissue was used for construction of CRC organoids. The study adhered to the recommendations outlined in the Requirements of the Ethical Review System of Biomedical Research Involving Human by Tianjin Union Medical Center Ethics Committee. All subjects were given a written informed consent in accordance with the Declaration of Helsinki.

A more detailed description of materials and methods is provided in the Supplemental Materials.

## Results

### SF3B3 is overexpressed in CRC

**Fig. 1:**
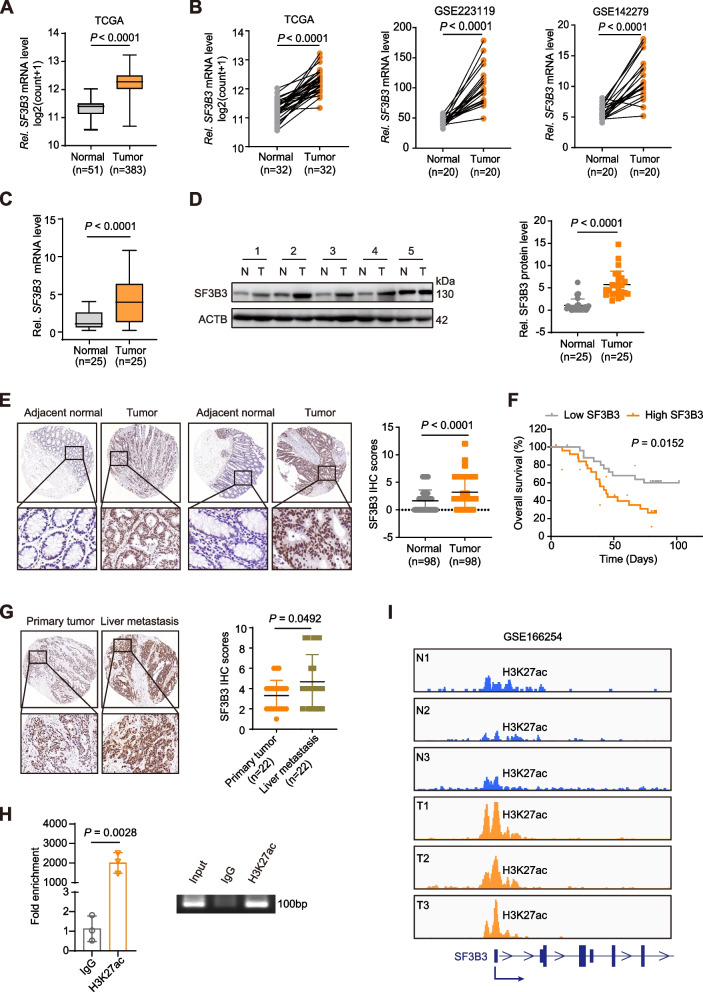
*SF3B3* expression is upregulated in human CRC. **A** *SF3B3* transcript levels in normal and CRC tissues. The data were obtained from TCGA-COAD and TCGA-READ datasets in Xena (http://xena.ucsc.edu/). **B** Relative *SF3B3* mRNA levels in CRC and pair matched adjacent normal tissues. The data were obtained from TCGA-COAD and TCGA-READ datasets in Xena, as well as GEO datasets. **C** Relative *SF3B3* mRNA levels in paired CRC and adjacent normal tissues. The mRNA expression was determined by qRT-PCR. **D** Western blot analysis of SF3B3 protein in paired CRC and adjacent normal tissues. **E** Representative IHC staining images of SF3B3 protein and statistical analysis of SF3B3 IHC scores in CRC and adjacent normal tissues. The staining extent was scored on a scale of 0 − 4. The staining intensity was scored at 0–3 (3 is the highest positivity). The final IHC score was generated by multiplying the score of staining extent with the score of staining intensity. The maximum score is 12. **F** Kaplan–Meier analysis of overall survival from CRC patients with high and low SF3B3 protein levels (*n* = 29). **G** Representative IHC staining images of SF3B3 protein and statistical analysis of SF3B3 IHC scores in primary CRC and their corresponding liver metastasis tissues. The final IHC score was generated by multiplying the score of staining extent with the score of staining intensity. **H** ChIP-PCR analysis of the enrichment of H3K27ac in *SF3B3* promoter region relative to IgG in LoVo cells. The immunoprecipitated DNA fragments by anti-H3K27ac and IgG were purified and analyzed by PCR with primers specific for *SF3B3* promoter. **I** Genome browser view of H3K27ac ChIP-seq data in *SF3B3* promoter region from CRC patient tissues. Data are shown as mean ± SD

The mRNA expression of SF3b components (*SF3B1-SF3B7*) was analyzed using The Cancer Genome Atlas-colonic adenocarcinoma (TCGA-COAD) and rectal adenocarcinoma (TCGA-READ) datasets. Consistent with a previous study [14], we observed significantly elevated mRNA levels of both *SF3B3* and *SF3B7* in CRCs (*n* = 383) compared to surrounding normal tissues (*n* = 51) (Fig. 1A and Fig. S1A). Notably, *SF3B3* mRNA levels were substantially higher than *SF3B7* mRNA levels in CRC tissues (Fig. S1B). Furthermore, only *SF3B3* mRNA expression was significantly upregulated in CRC tissues when normal colon tissues from the Genotype-Tissue Expression (GTEx) datasets were integrated with TCGA datasets (Fig. S1C). *SF3B3* mRNA levels were also higher in CRC tumors than their matched-paired normal tissues according to the analysis of samples from TCGA datasets and two recently published Gene Expression Omnibus (GEO) datasets (Fig. 1B).

We then explored the correlation of *SF3B3* expression with other genes using TCGA datasets. A total of 1574 genes showed positive co-expression with *SF3B3*, while 993 genes exhibited negative co-expression with *SF3B3* (|*r*|> 0.2, *p* < 0.05) (Tables S1 and S2). KEGG pathway enrichment analysis revealed that *SF3B3* positively co-expressed genes were most significantly enriched in the “spliceosome” pathway, whereas *SF3B3* negatively co-expressed genes were predominantly enriched in the “metabolic” pathway (Fig. S1D). Notably, *SF3B3* expression demonstrated a positive correlation with *MKI67*, which encodes a proliferation marker for tumor cells (Fig. S1E).

To validate the dataset analysis results, we quantified SF3B3 expression using qRT-PCR and western blot in 25 paired human CRC and adjacent normal tissues (Fig. 1C-D). Additionally, we performed immunohistochemical (IHC) analysis of SF3B3 in a cohort of 98 paired CRC and matched adjacent normal tissues (Fig. 1E). All these data supported the upregulation of *SF3B3* expression in CRC tissues. Kaplan–Meier analysis revealed that CRC patients with high SF3B3 levels had significantly reduced overall survival compared to those with low SF3B3 levels (Fig. 1F). Interestingly, SF3B3 protein levels in liver metastatic lesions were significantly higher than those in the matched primary CRC tissues (*n* = 22) (Fig. 1G). These clinical data strongly associate *SF3B3* expression with CRC metabolism, RNA splicing, proliferation, progression and prognosis.

As epigenetic modification was shown to be involved in the regulation of *SF3B7* expression [14], we investigated whether *SF3B3* expression was also epigenetically regulated. The Cistrome DB Toolkit (http://dbtoolkit.cistrome.org) was employed to analyze potential epigenetic factors for *SF3B3 *[16]. Among all histone modifications, histone H3-lysine-27 (H3K27ac) exhibited the highest regulatory potential score across all chromatin immunoprecipitation sequencing (ChIP-seq) samples (Fig. S1F). H3K27ac was found to be notably enriched in the promoter of *SF3B3*, as determined using the UCSC Genome Bioinformatics Site (http://genome.ucsc.edu/) (Fig. S1G). ChIP-seq data analysis in CRC cells also identified H3K27ac occupancy in the promoter region of *SF3B3* (Fig. S1H). Furthermore, we confirmed this occupancy through ChIP-PCR in CRC cells (Fig. 1H). Treatment with curcumin, an inhibitor of histone acetylation, was able to downregulate the expression of *SF3B3*, as well as a known H3K27ac-targeted gene *FOXM1* (Fig. S1I). ChIP-seq data analysis of CRC patient tissues (GEO: GSE166254) revealed that the H3K27ac peak in *SF3B3* promoter region was significantly higher in tumors than in normal tissues (Fig. 1I). These findings suggest that the upregulation of *SF3B3* in CRC tissues is at least partially attributed to promoter histone acetylation.

### SF3B3 promotes CRC proliferation, migration and invasion in vitro

**Fig. 2:**
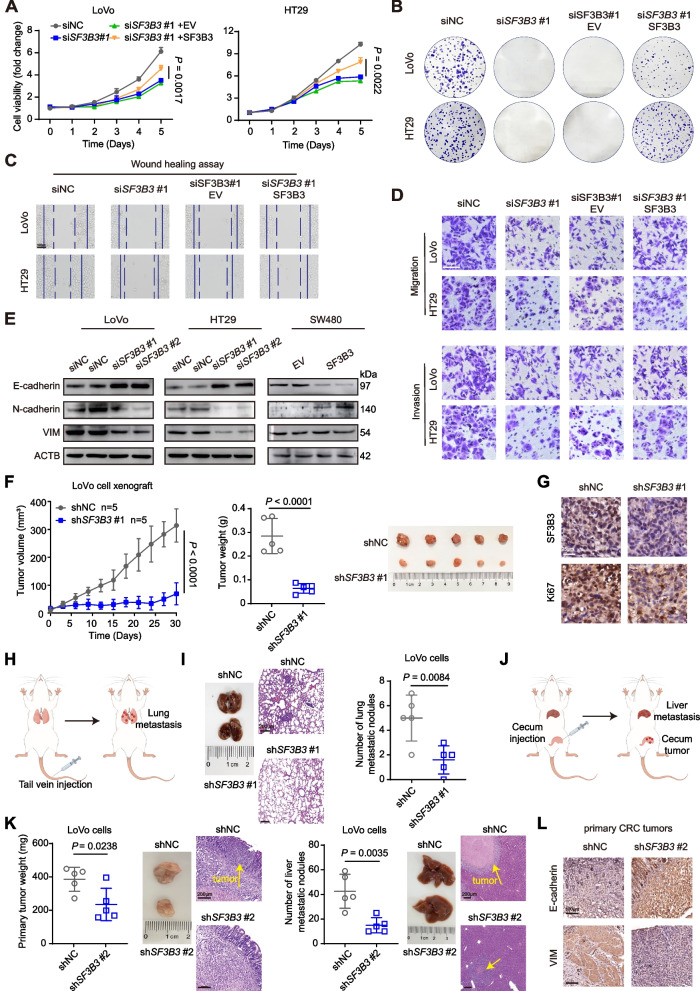
SF3B3 promotes the proliferation and metastasis of CRC in *Vitro* and *Vivo*.** A** Growth curves and (**B**) colony formation of LoVo and HT29 cells after *SF3B3* knockdown by siRNAs (siNC vs si*SF3B3*), or after *SF3B3* knockdown by siRNAs for 12 h followed with re-expression of *SF3B3* (si*SF3B3* + EV vs si*SF3B3* + SF3B3) (empty vector, EV; SF3B3 overexpressing plasmid, SF3B3). Cell viability was determined at different time points and colony formation assay was measured after 2 weeks. **C** Wound healing assays and (**D**) Transwell assays of LoVo and HT29 cells to investigate the effects of *SF3B3* knockdown (siNC vs si*SF3B3*) or re-expression (si*SF3B3* + EV vs si*SF3B3* + SF3B3) on cell migration and invasion abilities. Scale bars, 100 μm. **E** Representative western blots of EMT-related proteins in *SF3B3*-knockdown or *SF3B3*-overexpressing CRC cells. **F** Tumor growth, tumor weights, and tumor images of xenografts in nude mice. LoVo cells were infected with shNC or sh*SF3B3*#1 lentivirus to obtain the stably cell clones, which were subcutaneously injected into flank region of each nude mouse. **G** Representative IHC images of SF3B3 and Ki67 proteins in xenografts. Scale bars, 25 μm. **H** Schematic design of the CRC lung metastasis mouse model. **I** Representative images of lung, H&E staining of lung tissues derived from mice after tail vein injection with stably *SF3B3*-knockdown LoVo cells (LoVo-sh*SF3B3*#1), and statistical analysis of lung metastatic nodules. Scale bars, 200 μm. **J** Schematic design of the CRC liver metastasis mouse model. **K** Statistical analysis of primary CRC tumor weights and liver metastatic nodules, as well as representative H&E staining. Stably *SF3B3*-knockdown LoVo cells were constructed using sh*SF3B3*#2 lentivirus. Scale bars, 200 μm. **L** Representative IHC images of E-cadherin and VIM in primary CRC tumors from mice orthotopically injected with *SF3B3*-knockdown LoVo cells (LoVo-sh*SF3B3*#2). Scale bars, 100 μm. Data are presented as mean ± SD

The functional roles of SF3B3 were determined in CRC cell lines. We found that *SF3B3* mRNA and protein levels were generally higher in CRC cells compared to normal human mucosal epithelial cells (Fig. S2A). LoVo and HT29 cells with high *SF3B3* expression were selected for knockdown experiments. Two *SF3B3* specific siRNAs were identified, and their knockdown effects were validated at both mRNA and protein levels (Fig. S2B). Both *SF3B3* siRNAs significantly suppressed cell viability (Fig. 2A, Fig. S2C) and colony formation (Fig. 2B, Fig. S2D) of LoVo and HT29 cells, which were largely rescued by *SF3B3* re-expression. Conversely, *SF3B3* overexpression promoted cell growth and colony formation of SW480 cells that had low endogenous *SF3B3* expression (Fig. S2B, E).

We then determined the impact of SF3B3 on the invasive and migratory ability of CRC cells using Wound healing scratch assays and Transwell assays. *SF3B3* siRNAs markedly suppressed the migration and invasion capacity of LoVo and HT29 cells, which could be rescued by *SF3B3* re-expression (Fig. 2C-D, Fig. S2F-H). Epithelial-mesenchymal transition (EMT) is an important event during cancer invasion and metastasis. Western blot and immunofluorescence staining analyses revealed that *SF3B3* knockdown enhanced the level of the epithelial marker E-cadherin, while reducing the levels of the mesenchymal markers vimentin (VIM) and N-cadherin (Fig. 2E, Fig. S2I). Conversely, *SF3B3* overexpression exerted the opposite effects on EMT markers. These findings indicate that SF3B3 promotes the proliferation and metastasis of CRC cells in vitro.

### Silencing of SF3B3 impedes CRC proliferation and metastasis in vivo

To investigate the in vivo function of SF3B3, we utilized a lentivirus-mediated shRNA system to construct stably *SF3B3*-knockdown CRC cells (LoVo-sh*SF3B3*#1, LoVo-sh*SF3B3*#2 cells, HT29-sh*SF3B3*#2 cells) for mouse CRC models (Fig. S3A-B). Firstly, LoVo-sh*SF3B3*#1 and control (LoVo-shNC) cells were subcutaneously injected into nude mice for xenograft tumor formation. Compared to controls, the growth of LoVo-sh*SF3B3*#1 xenografts was significantly suppressed with no significant difference in mouse body weights (Fig. 2F, Fig. S3C). IHC analysis revealed that both SF3B3 and Ki67 immunoreactivities were significantly lower in LoVo-sh*SF3B3*#1 xenografts compared to controls (Fig. 2G). Moreover, LoVo-sh*SF3B3*#1 xenografts demonstrated an increase in E-cadherin immunoactivity alongside a reduction in VIM immunoactivity compared to controls (Fig. S3D). Secondly, we constructed a CRC lung metastasis models by tail vein injection of LoVo-sh*SF3B3*#1 or HT29-sh*SF3B3*#2 cells into nude mice (Fig. 2H). The number and size of metastatic lesions produced by LoVo-sh*SF3B3*#1 or HT29-sh*SF3B3*#2 cells were significantly decreased compared to their corresponding controls (Fig. 2I, Fig. S3E). Finally, we established a clinically relevant orthotopic mouse model by injecting control and LoVo-sh*SF3B3#2* cells into the cecum termini of NSG mice [17] (Fig. 2J). Interestingly, *SF3B3* knockdown not only suppressed the primary tumor growth in the cecum, but also inhibited the liver metastasis of LoVo cells (Fig. 2K). Compared to controls, the primary CRC tumor tissues formed in LoVo-sh*SF3B3#2* group demonstrated a stronger E-cadherin staining and much weaker VIM staining (Fig. 2L). Consistently, *SF3B3* knockdown led to increased E-cadherin staining and decreased VIM staining in both lung and liver metastasis tumors (Fig. S3F). Altogether, these findings suggest that SF3B3 promotes CRC proliferation and metastasis in *vivo*.

### Silencing of SF3B3 induces the intrinsic apoptosis pathway in CRC cells

**Fig. 3:**
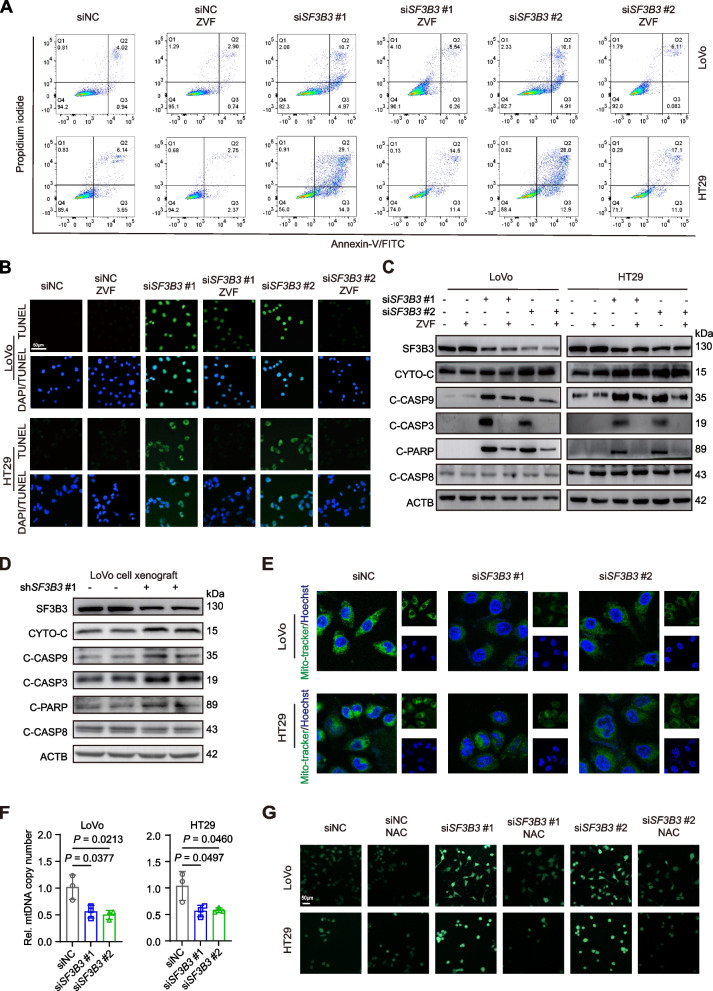
Silencing of *SF3B3* induces the intrinsic apoptotic pathway in CRC cells.** A** Detection of apoptosis by Annexin VFITC/PI staining and flow cytometric analysis. LoVo and HT29 cells were transfected with siRNAs for 48 h followed by 20 μM ZVF (Z-VAD-FMK) treatment for 24 h. **B** Representative images of TUNEL staining. Nuclei (blue) were stained with DAPI. CRC cells were transfected with siRNAs for 48 h followed by 20 μM ZVF treatment for 24 h. Scale bars, 50 μm. **C** Representative western blots of apoptosis-related proteins in CRC cells. LoVo and HT29 cells were transfected with siRNAs for 48 h, followed by 20 μM ZVF treatment for 24 h. **D** Representative western blots of apoptosis-related proteins in LoVo xenografts (shNC vs shSF3B3#1). **E** Representative fluorescence images of MitoTracker (green) staining using a confocal microscope with 63 × oil immersion lens. Nuclei (blue) were stained with Hoechst 33342. LoVo and HT29 cells were treated with siRNAs for 72 h. **F** mtDNA copy number was quantified by qRT-PCR. LoVo and HT29 cells were treated with siRNAs for 48 h. Data are shown as mean ± SD. **G** Representative fluorescence images of ROS (green) staining. LoVo and HT29 cells transfected with siRNAs for 24 h, followed by 10 mM NAC (N-acetylcysteine) treatment for 48 h. Scale bars, 50 μm

To elucidate the mechanism underlying SF3B3-mediated regulation of CRC cell growth, we treated *SF3B3*-knockdown cells with ZVF (Z-VAD-FMK, a pan-caspase inhibitor of apoptosis), NSA (necrosulfonamide, an inhibitor of necroptosis), or ferroptosis inhibitors (Lip-1, liproxstatin-1; Fer-1, ferrostatin-1). Only the apoptosis inhibitor ZVF significantly restored the growth of *SF3B3*-knockdown cells (Fig. S4A). Flow cytometric analysis and terminal deoxynucleotidyl transferase-mediated dUTP-fluorescein nick end labeling (TUNEL) staining assays demonstrated a significant promotion of apoptosis in *SF3B3*-knockdown LoVo and HT29 cells, which could be rescued by ZVF (Fig. 3A-B).

There are two major pathways leading to apoptosis. The intrinsic apoptosis pathway is activated by the release of cytochrome C from mitochondria, leading to caspase-9 activation, whereas the extrinsic apoptosis pathway is characterized with caspase-8 activation. Western blot analysis revealed that *SF3B3* knockdown significantly increased the protein levels of cytochrome C, cleaved caspase-9, cleaved caspase-3, and cleaved poly ADP-ribose polymerase (PARP), but had no effect on cleaved caspase-8 (Fig. 3C). Such alterations were markedly rescued by ZVF. Furthermore, the levels of intrinsic apoptosis protein markers were also significantly increased in LoVo-sh*SF3B3* xenografts compared to controls (Fig. 3D).

Mitochondria injury and reactive oxygen species (ROS) are major contributors to the intrinsic apoptosis pathway [18]. *SF3B3* knockdown decreased mitochondrial mass in CRC cells, as revealed by Mito-Tracker staining and mitochondrial DNA (mtDNA) quantification (Fig. 3E-F). Additionally, ROS production was markedly elevated in *SF3B3*-knockdown CRC cells, which was restored by the ROS inhibitor N-acetyl cysteine (NAC) (Fig. 3G). NAC also markedly rescued the growth and restored the protein levels of apoptosis markers in *SF3B3*-knockdown CRC cells (Fig. S4B-C). Collectively, these findings suggest that silencing of *SF3B3* induces mitochondria-mediated intrinsic apoptosis.

### SF3B3 regulates lipogenesis in CRC cells via SREBF1-FASN signaling

**Fig. 4:**
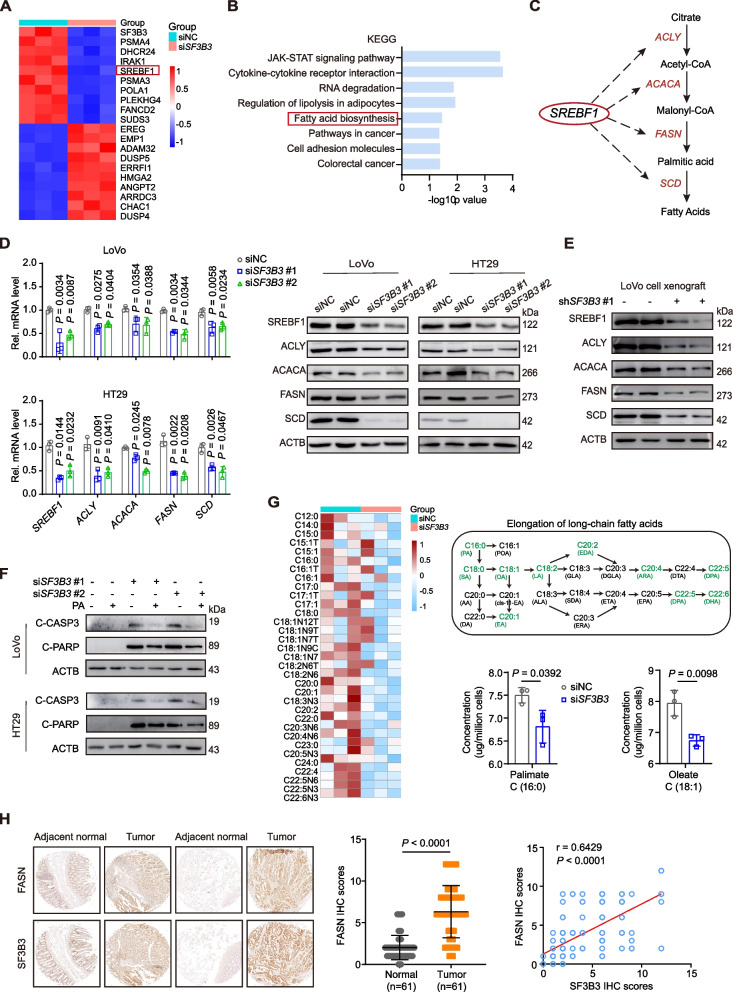
SF3B3 regulates lipogenesis in CRC cells via SREBF1-FASN signaling. **A** Heatmap displaying the top 10 up- and down-regulated genes from RNA-seq analysis of LoVo cells transiently transfected with siRNAs (si*SF3B3*#1 + si*SF3B3*#2) for 48 h. **B** KEGG pathway analysis of *SF3B3*-regulated genes from RNA-seq. **C** Schematic representation of SREBF1-dependent lipogenesis. **D** mRNA expression of *SREBF1* and its target genes, as well as representative western blots of the proteins in *SF3B3*-knockdown CRC cells. mRNA levels were detected at 48 h, and protein levels were detected at 72 h after transfection. **E** Representative western blots of SREBF1, ACLY, ACACA, FASN and SCD in LoVo xenografts (shNC vs sh*SF3B3*#1). **F** Representative western blots of C-CASP3 and C-PARP in *SF3B3*-knockdown CRC cells. Cells were transfected with siRNAs for 24 h, then treated with or without 10 μM PA (palmitate) for 48 h. **G** Lipidomic profiles displaying the fatty acids in LoVo cells transiently transfected with siRNAs (si*SF3B3*#1 + si*SF3B3*#2) for 72 h. Schematic representation of long-chain fatty acids that were significantly decreased (in green) in *SF3B3*-knockdown LoVo cells. **H** Representative IHC images of FASN in CRC tissues and adjacent normal tissues. The final IHC score was generated by multiplying the score of staining extent with the score of staining intensity. Statistical analysis of FASN IHC scores and correlation analysis with SF3B3 in CRC tissues. Data are shown as mean ± SD

To further elucidate the molecular mechanism by which SF3B3 regulated CRC proliferation and metastasis, we conducted RNA-seq of LoVo cells with or without *SF3B3* knockdown. A total of 715 significantly upregulated and 419 downregulated genes were identified following *SF3B3* knockdown (Table S3, Fig. S5A). As anticipated, *SF3B3* emerged as one of the top significantly downregulated genes by *SF3B3* knockdown (Fig. 4A, Fig. S5B). KEGG pathway analysis revealed that the differentially expressed genes due to *SF3B3* knockdown were enriched in various pathways, including the “Fatty acid biosynthesis” pathway (Fig. 4B). SREBF1 (sterol regulatory element binding transcription factor 1, also known as SREBP1) is a master transcriptional regulator for lipogenesis genes, such as *ACLY*, *ACACA*, *FASN* and *SCD *[19] (Fig. 4C). Interestingly, *SREBF1* stood out as one of the top 5 most significantly down-regulated genes by *SF3B3* knockdown (Fig. 4A, Table S3). The transcript levels of *SREBF1*, *ACLY*, *ACACA*, *FASN* and *SCD* were all significantly decreased by *SF3B3* knockdown (Fig. S5C). Such alterations were validated using both qRT-PCR and western blot assays (Fig. 4D). In contrast, *SF3B3* knockdown had no significant effect on *SREBF2*, which encodes another SREBP nuclear transcription factor for genes involved cholesterol biosynthesis (Fig. S5D). Importantly, the protein levels of SREBF1, ACLY, ACACA, FASN and SCD were also lower in LoVo-sh*SF3B3* xenografts compared to controls (Fig. 4E).

Lipid metabolism reprogramming through SREBF1-FASN axis plays a vital role in the regulation of CRC progression and metastasis [20–22]. We found that *SF3B3* knockdown significantly decreased triglyceride levels in CRC cells (Fig. S5E). This was confirmed by Nile red staining (Fig. S5F). FASN catalyzes the synthesis of palmitate (PA), which is the starting point for other fatty acids. We found that palmitate could significantly decrease apoptosis biomarkers and ROS in *SF3B3*-knockdown CRC cells (Fig. 4F, Fig. S5G). To gain further insights into the role of SF3B3 in CRC metabolism, we utilized mass spectrometry for targeted lipidomic analysis of *SF3B3*-knockdown cells. Lipidomic profiles revealed a significant decrease in fatty acids, particularly palmitate (C16:0) and its downstream fatty acids (C18:0, C18:1, C18:2, C20:1, C20:2, C20:4, C22:5, C22:6), in *SF3B3*-knockdown cells compared to controls (Fig. 4G, Fig. S5H and Table S4).

TCGA dataset analysis revealed that the mRNA expression of *SF3B3* was positively correlated with that of *ACLY*, *ACACA*, *FASN* and *SCD* in human CRC tissues (Fig. S5I). Considering the critical role of FASN in CRC development [23–25], we assessed FASN protein levels in 61 paired CRC and matched adjacent normal tissues through IHC. We found that FASN protein levels were positively correlated with SF3B3 protein levels in CRC tissues (Fig. 4H). Altogether, these findings suggest that SF3B3 promotes the malignant phenotype of CRC, at least partially, by regulating SREBF1-FASN-mediated lipogenesis.

### SF3B3 regulates SREBF1 via mTOR signaling

**Fig. 5:**
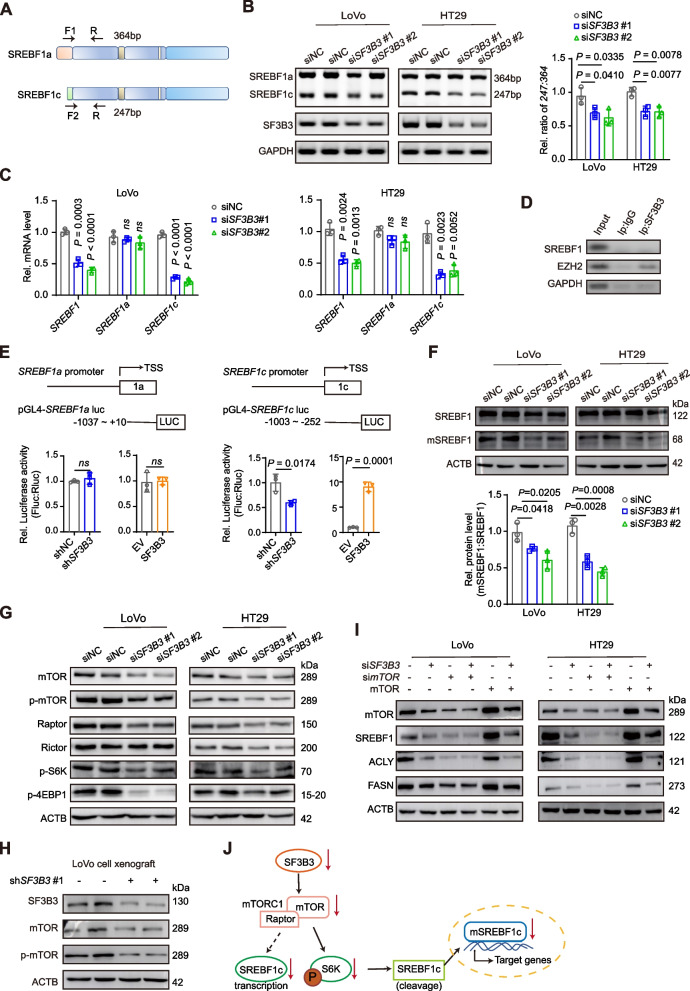
SF3B3 regulates SREBF1 via mTOR signaling. **A** Schematic representation of PCR primers used for the identification of *SREBF1a* and *SREBF1c*. **B** Relative *SREBF1a* and *SREBF1c* mRNA levels were examined and analyzed by 3% agarose gel electrophoresis of PCR products. LoVo and HT29 cells were treated with siRNAs for 48 h. **C** The mRNA expression of *SREBF1a* and *SREBF1c* was determined by qRT-PCR. **D** The immunoprecipitated RNAs by anti-SF3B3 and negative IgG were purified and analyzed by PCR using primers specific for *SREBF1* and *EZH2*. **E** Schematic diagram illustrating the *SREBF1a* and *SREBF1c* promoters. Dual luciferase reporter assay was used to determine *SREBF1a* and *SREBF1c* promoter activity in HEK293T cells after infection with sh*SF3B3*#1 lentivirus (shNC vs sh*SF3B3*) or transfection with overexpressing plasmids (empty vector, EV; SF3B3 overexpressing plasmid, SF3B3). **F** Western blot analysis of full-length SREBF1 and mature SREBF1 (mSREBF1) in *SF3B3*-knockdown CRC cells. LoVo and HT29 cells were treated with siRNAs for 72 h. **G** Representative western blots of mTOR, p-mTOR, Raptor, Rictor, p-S6K and p-4EBP1 in *SF3B3*-knockdown CRC cells. LoVo and HT29 cells were treated with siRNAs for 72 h. **H** Representative western blots of mTOR and p-mTOR in LoVo xenografts (shNC vs sh*SF3B3*#1). **I** Representative western blots of mTOR, SREBF1, ACLY, and FASN in *SF3B3*-knockdown CRC cells after *mTOR* silencing or overexpression. LoVo and HT29 cells were treated with siRNAs for 12 h, followed by transfection with either si*mTOR* or *mTOR*-overexpressing plasmids for 60 h. **J** Schematic diagram illustrating the role of mTOR in regulating SREBF1c in *SF3B3*-knockdown CRC cells. Data are shown as mean ± SD

SREBF1 comprises two isoforms, namely SREBF1a and SREBF1c, which are transcribed through alternative splicing at transcription start sites, but have distinct regulation of downstream target genes [26]. The two isoforms differ only in their first exon (exon 1a and exon 1c) (Fig. 5A). PCR analysis revealed that the decreased *SREBF1* mRNA in *SF3B3*-knockdown cells was due to the downregulation of *SREBF1c* mRNA (Fig. 5B-C). To assess whether SF3B3 is involved in the promoter splicing of *SREBF1*, we performed RNA immunoprecipitation (RIP) assays, using the known SF3B3 target gene *EZH2* as the positive control [27]. Notably, we did not detect *SREBF1* mRNA in the SF3B3 protein-antibody-bead system (Fig. 5D). This suggests that SF3B3-mediated regulation of *SREBF1c* expression does not stem from the alternative splicing of *SREBF1* promoter.

Next, we determined whether SF3B3 regulated *SREBF1* at the transcription or protein level. The promoter DNA fragments (~ 1000 bp upstream of the first exon) of *SREBF1a* and *SREBF1c* were constructed into dual luciferase report vectors. *SREBF1a* promoter activity was not altered by *SF3B3* knockdown or over-expression. In contrast, *SREBF1c* promoter activity was positively associated with *SF3B3* expression (Fig. 5E). At the protein level, SREBF1 activation involves the proteolytic cleavage of the N-terminal half of the protein followed by nuclear translocation. Intriguingly, *SF3B3* knockdown significantly decreased the ratio of mature SREBF1 to full-length SREBF1 in CRC cells (Fig. 5F). These findings suggest that SF3B3 regulate SREBF1c in CRC cells through both gene transcription and protein cleavage.

Because mTOR has been reported to regulate both *SREBF1c* transcription and activation [28–30], we determined whether mTOR was involved in SF3B3-mediated regulation of SREBF1c. Notably, *SF3B3* knockdown significantly decreased the protein levels of both p-mTOR and mTOR in CRC cells (Fig. 5G). Furthermore, LoVo-sh*SF3B3* xenografts also showed lower p-mTOR and mTOR levels compared to controls (Fig. 5H). mTOR is a critical component of two multiprotein signaling complexes named mTORC1 and mTORC2. Functionally, mTORC1 is considered a master regulator of cell growth and proliferation, whereas mTORC2 primarily regulates cytoskeletal structure and cell survival [31]. We found that *SF3B3* knockdown decreased the protein level of Raptor, a component of mTORC1, but had no significant effect on the protein level of Rictor, a component of mTORC2. Consistently, *SF3B3* knockdown decreased the protein levels of two mTORC1 downstream effectors, pS6K and 4EBP1 (Fig. 5G). Furthermore, we found that the downregulation of *SREBF1* and its target genes (*ACLY* and *FASN*) by *SF3B3* knockdown was not prominent in *mTOR*-knockdown cells, and could be largely restored by *mTOR* overexpression (Fig. 5I). The tuberous sclerosis protein complex (TSC complex) is a critical negative regulator of mTOR [32]. We found that knockdown of both *TSC1* and *TSC2* could significantly increase the mRNA and protein expression of *SREBF1*, *ACLY* and *FASN* in *SF3B3*-knockdown CRC cells (Fig. S5J-K). Collectively, these findings suggest that mTORC1 regulates the gene transcription and protein cleavage of SREBF1c in *SF3B3*-knockdown CRC cells (Fig. 5J).

### SF3B3 regulates mTOR splicing

**Fig. 6:**
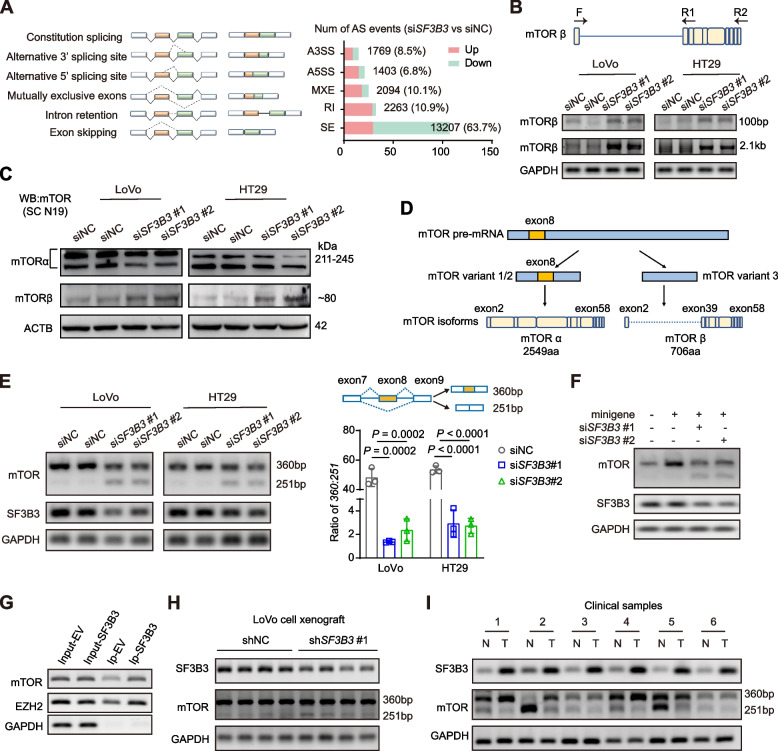
SF3B3 regulates mTOR exon 8 skipping in CRC cells. **A** Schematic diagram of alternative splicing types, and alternative splicing events regulated by *SF3B3* from RNA-seq data. **B** The *mTORβ* transcript levels were examined by 1% agarose gel electrophoresis of PCR products. The 2.1 kb product (full-length *mTORβ* transcript) was amplified with the primers (F, R2), whereas the 100 bp product (partial *mTORβ* transcript) was amplified with the primers (F, R1). **C** Representative western blots of full-length mTOR (mTORα) and its short-length isoform (mTORβ) in CRC cells using the mTOR antibody (sc-517464). LoVo and HT29 cells were treated with siRNAs for 72 h. **D** Schematic diagram of the protein-coding *mTOR* transcript variants and isoforms. *mTOR* variant 1 and variant 2 include exon 8, whereas *mTOR* variant 3 exclude exon 8 in NCBI dataset. Exon 8 is also skipped in *mTORβ* transcript. **E** Exon 8 skipping of *mTOR* was examined by 3% agarose gel electrophoresis of PCR products in CRC cells. LoVo and HT29 cells were treated with siRNAs for 48 h. Data are shown as mean ± SD. **F** Exon 8 skipping was determined by 3% agarose gel electrophoresis of PCR products in CRC cells after 48 h transfection with a minigene contains exons 7–9 as well as two introns of *mTOR*. **G** The immunoprecipitated RNAs by anti-SF3B3 or IgG were purified and analyzed by PCR using primers specific for *mTOR* and *EZH2*. *EZH2* used as positive control. **H** 3% agarose gel electrophoresis of PCR products of *mTOR* exon 8 skipping and *SF3B3* mRNA in control and *SF3B3*-knockdown LoVo xenografts (shNC vs sh*SF3B3*#1). **I** Representative agarose gel electrophoresis of PCR products of *mTOR* exon 8 skipping and *SF3B3* mRNA in paired CRC tissues and adjacent normal tissues

Because *SF3B3* knockdown significantly suppressed the mRNA expression of *mTOR* in CRC cells (Fig. S6A-B), we determined whether SF3B3 was involved in *mTOR* RNA splicing. Firstly, we assessed the impact of *SF3B3* knockdown on AS events in CRC cells by analyzing RNA-seq data. A total of 20,736 AS events were significantly altered by *SF3B3* knockdown, encompassing alternative 3’ splice site (ss) exon (A3SS), alternative 5’ ss exon (A5SS), mutually exclusive exons (MXE), retained intron (RI), and skipped exon (SE) events (Fig. 6A). Notably, more than 60% of these AS events corresponded to skipped exon (SE). KEGG pathway analysis indicated that *SF3B3*-regualted AS events were most significantly enriched in genes involved in “Metabolic pathways” and “Spliceosome”, which was consistent with the mRNA analysis using TCGA datasets (Fig. S6C). Secondly, we investigated whether *SF3B3* knockdown altered mTOR isoforms. To date, only two functional human mTOR isoforms, namely mTORα and mTORβ, have been identified [10, 33]. We used the same PCR primers and antibodies reported in previous study for detecting mTORβ (Fig. 6B, Fig. S6D). Strikingly, *SF3B3* knockdown increased both mRNA and protein levels of mTORβ in CRC cells (Fig. 6B-C). Thirdly, we analyzed the sequences of three protein-coding *mTOR* transcript variants (*mTOR* variant 1, NM_004958.4; *mTOR* variant 2, NM_001386500.1; *mTOR* variant 3, NM_004958.4) in NCBI database. *mTOR* variant 1 and variant 2 encode the same isoform, but differ from *mTOR* variant 3 at exon 8, where skipping occurs (Fig. 6D). Notably, exon 8 is also skipped in *mTORβ* transcript. We compared the rescue effects of full-length mTOR and mTOR variant 3 in *SF3B3*-knockdown CRC cells. Both cell viability and SREBF1 protein levels of *SF3B3*-knockdown cells could be significantly increased upon overexpression of full-length *mTOR* (+ exon 8), whereas overexpression of *mTOR* variant 3 (-exon 8) did not elicit a similar rescue effect (Fig. S6E). This suggests that exon 8-skipped splicing may attenuate the rescuing capability of mTOR in *SF3B3*-knockdown CRC cells.

We then investigated whether SF3B3 regulated exon 8 skipped splicing of *mTOR*. PCR analysis revealed that *SF3B3* knockdown markedly increased endogenous *mTOR* exon 8 skipping in CRC cells (Fig. 6E). The effect of *SF3B3* knockdown on exon 8 skipping was further validated in LoVo cells transfected with a minigene recombinant plasmid containing exons 7–9 of *mTOR* genomic DNA (Fig. 6F). We also examined the impact of *SF3B3* overexpression on *mTOR* splicing. As expected, *SF3B3* overexpression notably decreased *mTOR* exon 8 skipping in SW480 cells, with a concomitant decrease in mTORβ protein level (Fig. S6F). RIP analysis revealed that *mTOR* mRNA could be immunoprecipitated with the SF3B3 protein-antibody-bead system, which was higher in *SF3B3* overexpressing cells than in controls (Fig. 6G, Fig. S6G). Finally, we assessed the role of SF3B3 in regulating *mTOR* splicing in vivo. *SF3B3* knockdown increased exon 8 skipping of *mTOR* in LoVo cell xenografts (Fig. 6H). A higher *mTOR* exon 8 skipping was observed in tumor tissues compared to matched normal tissues, showing a negative correlation with *SF3B3* expression (Fig. 6I). Collectively, these findings suggest that SF3B3 regulates exon 8 skipped splicing of *mTOR* RNA.

### SF3B3 regulates autophagy

**Fig. 7:**
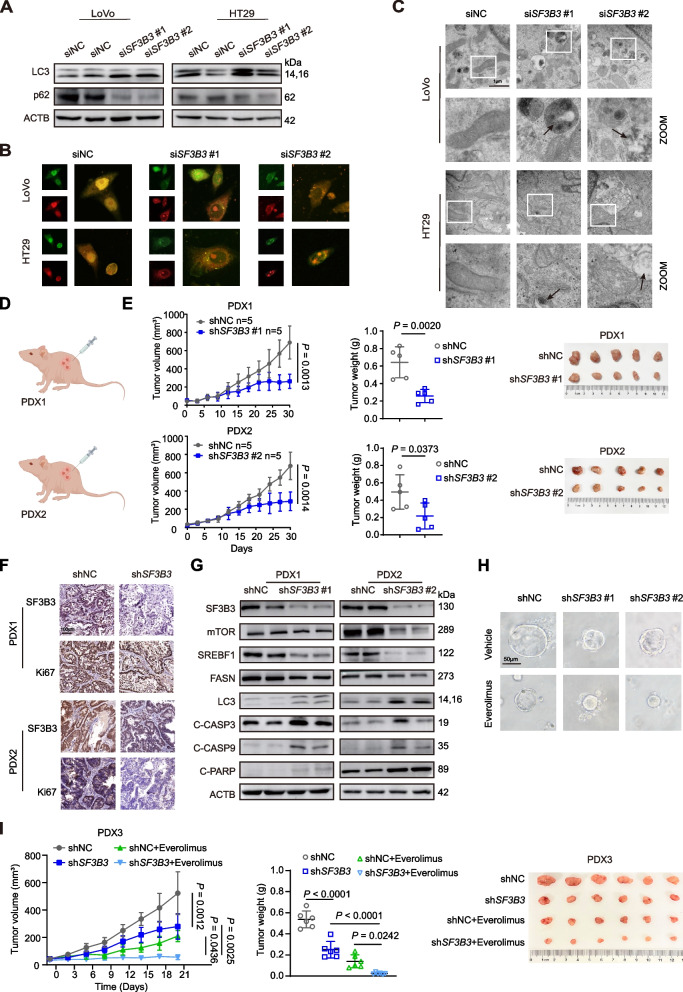
*SF3B3* knockdown and mTOR inhibitor induce synergistic antitumor activity. **A** Representative western blots of LC3 and SQSTM1/p62 in *SF3B3*-knockdown CRC cells. LoVo and HT29 cells were treated with siRNAs for 72 h. **B** Representative autophagic flux images. mCherry-GFP-LC3B labeled CRC cells were transfected with *SF3B3* siRNAs for 48 h. **C** Representative transmission electron microscopy images presenting the ultrastructure of the *SF3B3*-knockdown CRC cells. Arrows indicate autophagic vacuoles. LoVo and HT29 cells were treated with siRNAs (si*SF3B3*#1 + si*SF3B3*#2) for 72 h. Scale bars, 1 µm. **D** Schematic diagram of two PDX models. PDX1 models were treated Lentivirus-shNC vs Lentivirus-sh*SF3B3*#1, and PDX2 models were treated Lentivirus-shNC vs Lentivirus-sh*SF3B3*#2. **E** Tumor growth, tumor weights, and tumor photos of two PDX mouse models after intratumor injection of shNC or sh*SF3B3* lentivirus. **F** Representative IHC images of SF3B3 and Ki67 in two PDX tumor tissues. **G** Representative western blots of lipogenesis-, autophagy- and apoptosis-related proteins in PDX tumor tissues. **H** Representative images of patient-derived CRC tumor organoids after treatment with *SF3B3* shRNA lentivirus and everolimus. Digested organoids were transduced with sh*SF3B3* lentivirus for 6 h, followed by reconstitution in matrigel in 24-well plate. After culturing for 7 days, organoids were further treated with or without 40 μM everolimus for 48 h. Scale bars, 50 μm. **I** Tumor growth, tumor weights, and photos of PDX3 tumors. Mice were intratumorally injected with shNC or sh*SF3B3* lentivirus (sh*SF3B3*#1 + sh*SF3B3*#2), followed by oral gavage of vehicle (40%PEG400, 5% Tween-80 and 5% DMSO) or everolimus (5 mg/kg, p.o., every two days for 3 weeks). Data are shown as mean ± SD

Because mTOR is a master regulator of autophagy, we determined the impact of SF3B3 on autophagy. *SF3B3* knockdown in CRC cells significantly increased the accumulation of LC3B, a specific marker for autophagosomes, as revealed by both western blot and immunofluorescence analyses (Fig. 7A, Fig. S6H). The induction of LC3B in *SF3B3*-knockdown CRC cells could be restored by *mTOR* overexpression (Fig. S6I). *SF3B3* knockdown also significantly decreased the protein level of SQSTM1/p62, a marker for autophagic flux (Fig. 7A). By using the Lenti-mCherry-GFP-LC3B fluorescent assay, we found that *SF3B3* knockdown increased both autophagosomes and autophagolysosomes in CRC cells (Fig. 7B). Cloroquine (CQ) and Bafilomycin A1 (BafA1), two autophagy inhibitors that prevent the fusion of autophagosome with lysosome, significantly increased the accumulation of LC3B in *SF3B3*-knockdown CRC cells (Fig. S6J). By using transmission electron microscopy, we observed the autophagic vacuoles (double-membrane compartments containing lamellar structures) in *SF3B3*-knockdown CRC cells (Fig. 7C). These findings suggest that *SF3B3* knockdown induces autophagic flux in CRC cells.

Pan-cancer analysis of TCGA data revealed that *SF3B3* expression correlated positively with the expression of full-length *mTOR*-001 isoform (Fig. S6K). We then silenced *SF3B3* in breast cancer cells (MCF7), cervical cancer cells (Hela) and liver cancer cells (Huh7). Notably, *SF3B3* knockdown markedly increased exon 8 skipping of *mTOR* and LC3B protein levels in all three cancer cell lines (Fig. S6L-M). These findings suggest that SF3B3 plays a critical role in regulating mTOR splicing and autophagy in multiple cancers.

### SF3B3 knockdown and mTOR inhibitor induce synergistic antitumor activity

To assess the therapeutic potential of targeting SF3B3 in CRC, we constructed two CRC patient-derived xenograft (PDX) models. The clinical information of donor patients was provided in Fig. S7A. The two PDX models were administered sh*SF3B3*#1 and sh*SF3B3*#2 lentiviruses, respectively, via intratumor injection (Fig. 7D). *SF3B3* knockdown in two PDX murine models was validated by qRT-PCR (Fig. S7B). Compared to shNC control groups, the volume and growth rate of tumors in sh*SF3B3* groups were significantly decreased (Fig. 7E). IHC staining showed that Ki67 levels markedly decreased in sh*SF3B3* groups (Fig. 7F). A significant increase of apoptotic cells was observed in sh*SF3B3* groups using TUNEL assay (Fig. S7C). Western blot analysis revealed that *SF3B3* inhibition markedly reduced protein levels of mTOR, SREBF1 and FASN (Fig. 7G). Additionally, the autophagy- and apoptosis-related markers were significantly elevated in sh*SF3B3* groups compared to shNC groups.

**Fig. 8:**
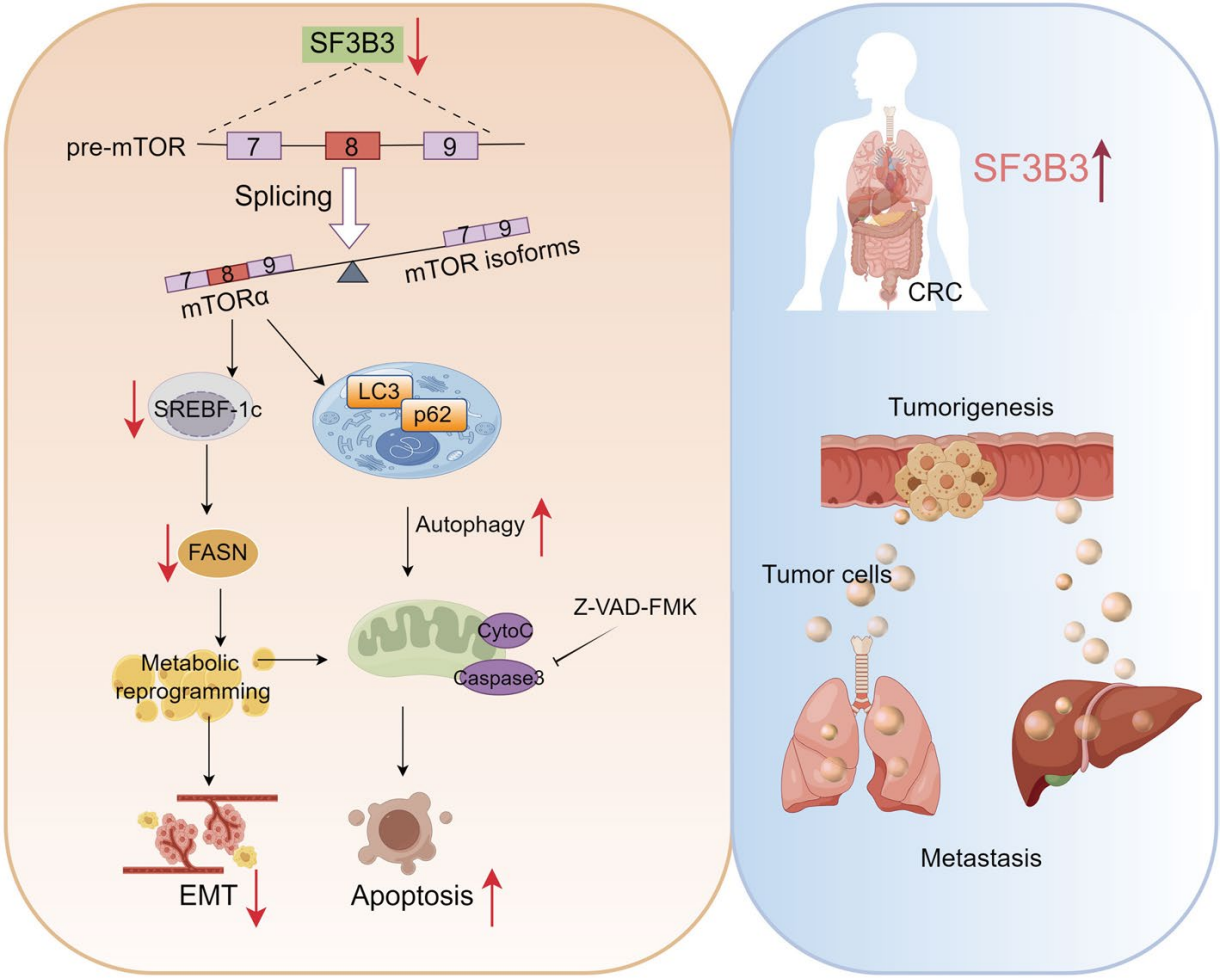
Mechanistic model illustrating the role of SF3B3 in CRC. SF3B3 is upregulated in CRC tissues, promoting CRC tumorigenesis and metastasis. Targeting SF3B3 through RNA silencing downregulates mTORα via alternative splicing, resulting in the induction of autophagy, suppression of *SREBF1* transcription, and inhibition of SREBF1 protein activation. Consequently, silencing of *SF3B3* prevents CRC progression and metastasis by inhibiting lipogenesis and promoting apoptosis

Notably, silencing of *SF3B3* significantly increased the sensitivity of two mTOR inhibitors, namely rapamycin and everolimus, in LoVo and HT29 cells (Fig. S7D). We then determined whether SF3B3 inhibition could be combined with mTOR inhibitors for CRC treatment in patient-derived CRC organoids (Fig. S7E). Interestingly, the combination of sh*SF3B3* lentivirus and everolimus markedly inhibited the CRC organoid growth (Fig. 7H). Furthermore, a third PDX mouse model was constructed, and mice were treated with sh*SF3B3* lentivirus by intratumor injection, followed by oral administration of everolimus. Notably, the combination treatment induced synergistically inhibitory effects on PDX growth, without affecting the body weights (Fig. 7I, Fig. S7F-G). Altogether, these findings suggest that SF3B3 is a potential therapeutic target for CRC treatment (Fig. 8).

## Discussion

The SF3b complex plays key roles in spliceosome assembly and activation. Among its seven components, SF3B1 is considered the most commonly mutated splicing factor across cancers [34]. The functions of SF3B1 and its inhibitors have been extensively evaluated in various cancers. In contrast, SF3B3 mutations are rare in cancers, and the functional role of SF3B3 in cancer development remains largely unknown. SF3B3 expression is upregulated in renal cancer, and is associated with tumor stage and poor prognosis[35]. Elevated expression of SF3B3 correlates with poor prognosis and tamoxifen resistance in ER-positive breast cancer [36]. Interestingly, knockdown of *SF3B3* inhibits the cell growth of both renal cancer cells and breast cancer cells. In this study, we demonstrate that SF3B3 is overexpressed in CRC, representing a potential therapeutic target for CRC treatment.

One of the major findings in this study is the critical role of SF3B3 in regulating alternative splicing of *mTOR* mRNA. Mechanistic studies on mTOR alternative splicing have been limited thus far. Src associated in mitosis (SAM68) was reported to promote splicing at the 5’ splice site in intron 5 of mTOR by interacting with U1 small nuclear ribonucleoprotein (U1 snRNP) [37, 38]. It should be noted that this short-length premature mTOR isoform is not functional. Various *mTOR* transcripts have been listed in different databases. A total of 7 mTOR isoforms are identified in TCGA datasets (mTOR-001 to mTOR-007) (Fig. S6I). The full-length *mTOR*-001 and the short-length *mTOR*-002 are highly expressed in tumors. Similar as functional mTORβ isoform, exon 8 is skipped in mTOR-002. In this study, we identify SF3B3 as a key mechanism for the formation of mTORβ and other exon 8-skipped mTOR isoforms. Additional research is necessary to explore the involvement of various mTOR isoforms in SF3B3-mediated progression and metastasis of CRC cells.

The findings in this study are significant for the development of mTOR inhibitors, which have been developed and evaluated in clinical trials for various cancer types. First, skipping exons in nucleotide sequence may lead to disruption of the drug-binding site, which may affect the efficacy and resistance of drugs. For instance, *mTORβ*-overexpressing cells is less sensitive to the inhibitory effect of rapamycin compared to mTORα-overexpressing cells [10]. Therefore, a better understanding of *mTOR* splicing may enhance the success of mTOR inhibitor development. Second, our results suggest a critical role of SF3B3 in regulating *mTOR* expression. A positive correlation between *SF3B3* and the full length* mTOR* isoform (*mTOR*-001) was identified in multiple cancers based on analysis of TCGA datasets. Therefore, mTOR inhibitors might be helpful in the treatment of *SF3B3* highly expressed cancers. Third, inhibiting multiple nodes of a pathway, either downstream or upstream of the driver oncogene, has proven a rational and promising approach for tumor therapy. Our findings suggest that combination treatment of both SF3B3 and mTOR inhibitors may represent a promising approach for CRC therapy.

This study identifies SF3B3 as a negative autophagic regulator across various cancers. Interestingly, knockdown of *SF3B3* was also shown to increase the expression levels of LC3B in neural cells [39]. This may suggest that SF3B3-regualted autophagy is not limited to tumor cells. A previous study reported that *SF3B3* knockdown induces autophagy in breast cancer cells, but the underlying mechanism is not fully elucidated [40]. This study demonstrates that SF3B3-regualted mTOR splicing contributes to autophagy in *SF3B3*-knockdown breast cancer cells. Autophagy plays context-dependent roles in various types of cancers [41]. In breast cancer cells, autophagy induced by *SF3B3* knockdown inhibits the proliferation and migration of breast cancer cells without inducing apoptosis [40]. Consistently, autophagy inhibitors show little effect on cell death (apoptosis) of *SF3B3*-knockdown CRC cells (data not shown). Future studies are needed to elucidate the exact role of autophagy in *SF3B3*-knockdown CRC cells.

Lipid metabolism reprogramming is a hallmark of cancer cells. In normal epithelial cells, fatty acids can be obtained from diet or synthesized de novo from carbohydrate precursors. In contrast, most fatty acids in tumor cells are derived from de novo lipogenesis [42]. SREBF1 and FASN are two central regulators of lipogenesis. SREBF1 mainly regulates the transcription of lipogenic genes. FASN is the key rate-limiting enzyme in de novo lipogenesis and catalyzes the formation of palmitate, the first fatty acid product in de novo lipogenesis. Both SREBF1 and FASN are significantly upregulated in CRC tissues compared with normal tissues [21, 43]. Inhibition of SREBF1 or FASN has been shown to inhibit CRC cell growth [20, 44]. Interestingly, SREBF1 is associated with the invasion and metastasis of CRC cells [21], whereas FASN plays a key role in regulating cell respiration [44]. This study suggests a critical role of SF3B3 in regulating lipid metabolism in CRC cells via SREBF1-FASN axis.

As *SF3B3* knockdown leads to alterations in numerous splicing events, it is unlikely that a single splicing event fully explains the role of SF3B3 in CRC. For instance, the increased ROS in *SF3B3*-knockdown CRC cells could be a consequence of multiple pathways, such as SF3B3-DHCR24 axis. Our RNA-seq data showed that *DHCR24* was among the top 5 most significantly down-regulated genes by *SF3B3* knockdown in CRC cells (Table S3). The downregulation of *DHCR24* expression in CRC cells by *SF3B3* knockdown was validated using qRT-PCR and western blot (Fig. S8A). Further study showed that *SF3B3* knockdown promoted the retention of intron between exon 3 and 4 of *DHCR24*, indicating a critical role of SF3B3 in regulating DHCR24 splicing (Fig. S8B). DHCR24 is reported to function as a hydrogen peroxide scavenger and plays an oncogenic role in various cancers [45, 46]. We found that reinforced expression of *DHCR24* significantly promoted cell growth and decreased the ROS production level of *SF3B3*-knockdown CRC cells (Fig. S8C-D). Furthermore, splicing factors such as SR and hnRNP proteins have been reported to regulate all levels of apoptotic gene expression, including transcription, alternative splicing, mRNA stability, translation, and protein stability [47]. We evaluated the effect of *SF3B3* knockdown on several critical apoptotic genes that have been previously documented to undergo alternative splicing events [48]. Notably, *SF3B3* knockdown increased the formation of pro-apoptotic mRNA isoform of *MCL1* (encoding myeloid cell leukemia-1) and *CASP9* (encoding caspase-9), both crucial genes involved in the intrinsic pathway of apoptosis (Fig. S8E). Therefore, it is likely numerous SF3B3 targets contribute to its phenotypic effects (such as apoptosis), and depletion of *SF3B3* could impact on multiple different pathways.

## Conclusion

Our in vitro and in vivo investigations demonstrated the importance of SF3B3 in promoting CRC progression and metastasis through regulating mTOR splicing and lipid metabolism (Fig. 8). Targeting SF3B3 by RNA silencing showed synergistic antitumor effects when combined with mTOR inhibitors. Additional studies in other cancer types may be investigated to test whether targeting SF3B3 could be broadly applied.

### Supplementary Information

**Additional file 1.** Supplementary materials and methods.

**Additional file 2: Table S1.** Positively TCGA. **Table S2.** Negatively TCGA. **Table S3.** RNA-Seq. **Table S4.** Lipidomics.

**Additional file 3.** Supplementary figures and tables. **Figure S1.** *SF3B3* is upregulated in human CRC and regulated by H3K27ac. (A) SF3B7 transcript levels in normal and CRC tissues. TCGA-COAD and TCGA-READ datasets (normal, *n*=51; cancer, *n*=383) were obtained from Xena (http://xena.ucsc.edu/). Data are shown as mean ± SD. (B) mRNA levels of SF3b family members in CRC tissues from TCGA-COAD and TCGA-READ datasets in cBioPortal (https://www.cbioportal.org/). (C) mRNA levels of SF3b family members in normal and CRC tissues after integrating the data of normal colon tissues from GTEx datasets with TCGA datasets. Analysis was performed by GEPIA2 (http://gepia2.cancer-pku.cn/). (D) KEGG pathway analysis of *SF3B3* positively and negatively co-expressed genes that were identified using cBioPortal (https://www.cbioportal.org/). (E) Correlation analysis between *SF3B3* and *MKI67* mRNA levels in TCGA datasets from Xena (http://xena.ucsc.edu/). (F) Potential epigenetic factors for *SF3B3* gene using a parameter of 1-kb regulatory potential decay in Cistrome DB Toolkit (http://dbtoolkit.cistrome.org). (G) Visualization of H3K27ac enrichment in the promoter region of *SF3B3* gene using UCSC Genome Browser (http://genome.ucsc.edu/). (H) Genome browser view of H3K27ac occupancy in *SF3B3* promoter region. ChIP-Seq data from three CRC cell lines (GSE83968, GSE96069, and GSE71510) were retrieved from GEO datasets (http://www.ncbi.nlm.nih.gov/geo). (I) *SF3B3* mRNA levels in LoVo and HT29 cells treated with 40 μM curcumin for 24 h. FOXM1 was used as positive control for H3K27ac target. Data are shown as mean ± SD. **Figure S2.** SF3B3 promotes proliferation and metastasis in vitro. (A) qRT-PCR quantification of *SF3B3* mRNA expression as well as representative western blots of SF3B3 in 7 CRC cell lines and normal human colon mucosal epithelial cell line (NCM460). (B) qRT-PCR analysis of *SF3B3* mRNA as well as representative western blots of SF3B3. LoVo and HT29 cells were transfected with siRNAs and collected at 48 h for mRNA detection or at 72 h for protein detection. SW480 cells were transfected with *SF3B3*-overexpressing plasmids for 72 h. (C) Growth curves and (D) colony formation of LoVo and HT29 cells after SF3B3 knockdown by siRNAs (siNC vs siSF3B3#2), or after SF3B3 knockdown by siRNAs for 12 h followed with re-expression of SF3B3 (si*SF3B3*#2+EV vs si*SF3B3*#2+SF3B3) (empty vector, EV; SF3B3 overexpressing plasmid, SF3B3). (E) Growth curves and colony formation of SW480 cells after transfection with *SF3B3* overexpressing plasmids. (F) Wound healing assays and (G) Transwell assays were used to measure cell migration and invasion abilities. LoVo and HT29 cells were treated with siRNAs (siNC vs si*SF3B3*#2) to knockdown *SF3B3*. For *SF3B3* re-expression study, cells were treated with siRNAs (siNC vs si*SF3B3*#2) for 12 h, followed by transfection with EV or *SF3B3* overexpressing plasmids (si*SF3B3*#2+EV vs si*SF3B3*#2+SF3B3). Scale bars, 100 μm. (H) Statistical analysis of Wound healing and Transwell assay results. Data are shown as mean ± SD. (I) Representative immunofluorescence staining images of E-cadherin (green), VIM (red) and DAPI (blue) in CRC cells using a confocal microscope with 100× oil immersion lens. **Figure S3.** Silencing of *SF3B3* impedes CRC proliferation and metastasis in vivo. qRT-PCR and western blot analyses of stably *SF3B3*-knockdown LoVo (A) and HT29 (B) cells. LoVo cells were infected with sh*SF3B3*#1 or sh*SF3B3*#2 lentivirus, whereas HT29 cells were infected with sh*SF3B3*#2 lentivirus. (C) Body weights of LoVo cell xenograft nude mice. The stably *SF3B3*-knockdown LoVo cells (LoVo-sh*SF3B3*#1) were subcutaneously injected into flank region of nude mice. (D) Representative IHC images of E-cadherin and VIM proteins in LoVo-shNC and LoVo-sh*SF3B3*#1 xenografts. Scale bars, 25 μm. (E) Representative images of lung, H&E staining for lung tissues, and statistical analysis of metastatic nodules in lung (*n*=5/group). Control and *SF3B3*-knockdown HT29 cells (sh*SF3B3*#2) were injected into nude mice via the tail vein. Scale bars, 200 μm. Data are shown as mean ± SD. (F) Representative IHC images of E-cadherin and VIM proteins in metastasis lung and liver tissues. Scale bars, 200 μm. **Figure S4.** SF3B3 regulates mitochondria-mediated apoptosis in CRC cells. (A) Cell viability of *SF3B3*-knockdown CRC cells co-treated with apoptosis-, necrosis- or ferroptosis-inhibitors. LoVo and HT29 cells were transfected with siRNAs for 24 h, followed by treatment with 20 μM ZVF (Z-VAD-FMK), 10 μM NSA (necrosulfonamide), 1 μM Lip-1 (liproxstatin-1) or 1 μM Fer-1 (ferrostatin-1) for 48 h. (B) Cell viability of *SF3B3*-knockdown CRC cells co-treated with ROS inhibitor. LoVo and HT29 cells were transfected with siRNAs for 24 h, followed by treatment with 10 mM NAC (N-acetylcysteine) for 48 h. (C) Representative western blots of apoptosis-related proteins. LoVo and HT29 cells were transfected with siRNAs for 24 h, followed by 10 mM NAC for 48 h. Data are shown as mean ± SD. **Figure S5.** SF3B3 regulates lipogenesis in CRC cells via SREBF1-FASN signaling. (A) Heatmap and (B) volcano plot displaying the significantly differential expression genes from RNA-seq of LoVo cells after transfection with siRNAs (mixture of si*SF3B3*#1 and si*SF3B3*#2) for 48 h. (C) Transcript levels of lipogenesis-related genes from RNA-seq of control and *SF3B3*-knockdown LoVo cells. (D) Transcript levels of *SREBF2* in RNA-seq analysis of *SF3B3*-knockdown LoVo cells, as well as qRT-PCR analysis of SREBF2 mRNA in CRC cells after transfection with siRNAs for 48 h. (E) Triglyceride levels in SF3B3-knockdown CRC cells after transfection with siRNAs for 72 h. (F) Nile red staining (Red) images and analysis of *SF3B3*-knockdown CRC cells after transfection with siRNAs for 72 h. Nuclei (blue) were stained with DAPI. Scale bars, 50 μm. (G) Representative fluorescence images of ROS (green) staining. CRC cells were transfected with siRNAs for 24 h, followed by treatment with 10 μM palmitate for 48 h. Scale bars, 50 μm. (H) Concentrations of individual fatty acids in LoVo cells after transfection with siRNAs (mixture of siSF3B3#1 and si*SF3B3*#2) for 72 h. The data are calculated based on lipidomic study. (I) Correlation analysis of the mRNA expression between *SF3B3* and lipogenesis-related genes based on TCGA-COAD and TCGA-READ datasets using GEPIA2. (J) qRT-PCR quantification of the mRNA expression of SREBF1 and its target genes. (K) Representative western blots of p-S6K, SREBF1, ACLY, and FASN. LoVo and HT29 cells were transfected with siRNAs (mixture of siSF3B3#1 and siSF3B3#2) for 12 h, followed by transfection with combined si*TSC1* and si*TSC2* for 60 h. Data are shown as mean ± SD. **Figure S6.** SF3B3 regulates mTOR splicing and autophagy. (A) mRNA levels of mTOR in *SF3B3*-knockdown LoVo cells from RNA-seq data. (B) mRNA expression of *mTOR* was quantified by qRT-PCR in CRC cells after transfection with siRNAs for 48 h. (C) KEGG pathway enrichment analysis of SF3B3-regualted AS events from RNA-seq data. Differentially alternative splicing patterns were determined using the rMATS tool. (D) Representative western blots of mTORα and mTORβ in the protein extracts of HEK293T cells using previous reported N-terminal mTOR antibody (sc-517464). (E) Cell viability and representative western blots of mTOR and SREBF1 of *SF3B3*-knockdown CRC cells after mTOR (+exon8 or -exon8) overexpression. LoVo and HT29 cells were transfected with si*SF3B3*#1 for 12 h, followed by transfection with overexpressing plasmids for full length mTOR (+exon 8) or mTOR variant 3 (-exon 8) for 60 h. (F) Exon 8 skipping of mTOR was examined by 3% agarose gel electrophoresis of PCR products in SW480 cells. Representative western blots of mTORα and mTORβ in SW480 cells using the mTOR antibody (sc-517464). SW480 cells were transfected with *SF3B3*-overexpressing plasmids for 72 h. Data are shown as mean ± SD. (G) RNA-IP analysis of the enrichment of mTOR in SF3B3 protein-antibody-beads system in LoVo cells. (H) Representative immunofluorescence images of LC3B (green) and DAPI (blue) in *SF3B3*-knockdown CRC cells using a confocal microscope with 63× oil immersion lens. LoVo and HT29 cells were transfected with siRNAs for 72 h. (I) Representative western blots of LC3B in *SF3B3*-knockdown CRC cells after forced expression of mTOR. LoVo and HT29 cells were transfected with siRNAs for 12 h, followed by transfection with mTOR-overexpressing plasmids for 60 h. (J) Representative western blots of LC3 in *SF3B3*-knockdown CRC cells after treatment with autophagy inhibitors. LoVo and HT29 cells were transfected with siRNAs for 24 h, followed by treatment with 20 μM CQ or 80 nM BafA1 for 48 h. (K) mTOR isoform structures identified in TCGA datasets (illustrated by GEPIA2). Correlation analysis between the SF3B3 mRNA levels and full-length mTOR-001 based on BRCA-, CESC- and LIHC-TCGA datasets using GEPIA2. (L) Exon 8 skipping of mTOR was examined by 3% agarose gel electrophoresis of PCR products in *SF3B3*-knockdown MCF7, Hela and Huh7 cells transfected with siRNAs for 48 h. (M) Representative western blots of mTOR and LC3 in *SF3B3*-knockdown MCF7, Hela and Huh7 cells transfected with siRNAs for 72 h. Data are shown as mean ± SD. **Figure S7.** Effect of *SF3B3* knockdown and mTOR inhibitor on CRC. (A) The clinical information of three donor patients investigated in this study. (B) qRT-PCR verification of *SF3B3* knockdown in two PDX murine models. PDX1 models were intratumorally treated with sh*SF3B3*#1 lentivirus, whereas PDX2 models were treated with sh*SF3B3*#2 lentivirus. (C) Representative H&E staining and TUNEL staining (green) of two PDXs. Scale bars, 50 μm. (D) Cell viability of LoVo and HT29 cells after transfection with siRNAs for 24 h, followed by rapamycin or everolimus treatment at the indicated concentrations for 48 h. (E) Viability and qRT-PCR validation of *SF3B3*-knockdown CRC tumor organoids. Digested organoids were transduced with shSF3B3 lentivirus for 6 h, followed by reconstitution in matrigel for 7 days. (F) Body weights of PDX mice. Xenograft tumors were transplanted into left and right flanks of each nude mouse to establish PDXs. Mice were intratumorally injected with shNC lentivirus on the left flank and sh*SF3B3* lentivirus (mixture of sh*SF3B3*#1 and sh*SF3B3*#2) on the right flank. After that, mice were orally administered vehicle (40%PEG400, 5% Tween-80 and 5% DMSO) or everolimus (5 mg/kg, every two days for 3 weeks). (G) Representative H&E staining as well as immunohistochemistry images of mTOR and Ki67 in PDX3 tumor tissues. Data are shown as mean ± SD. **Figure S8.** SF3B3 regulates alternative splicing of *DHCR24* and apoptotic genes. (A) mRNA and protein levels of *DHCR24*. CRC cells were transfected with siRNAs, and collected at 48 h for qRT-PCR and at 72 h for western blots. (B) Minigene detection showed the effects of *SF3B3* knockdown on *DHCR24* splicing in CRC cells. The minigene contains exons 3-4 of *DHCR24*. LoVo cells were transfected with *DHCR24* minigene together with *SF3B3* siRNAs for 48 h. (C) Detection of cell viability and (D) ROS in stably *SF3B3*-knockdown CRC cells transfected with *DHCR24* overexpressing plasmid for 72 h. Data are shown as mean ± SD. (E) Alternative splicing of apoptotic genes was examined by 1-3% agarose gel electrophoresis of PCR products in CRC cells. LoVo and HT29 cells were transfected with siRNAs for 48 h. **Table S5.** Sequences of siRNAs. **Table S6.** Sequences for plasmid construction. **Table S7.** Sequences of shRNAs. **Table S8.** Primer sequences for qRT-PCR or PCR. **Table S9.** List of antibodies used in this study.

## Data Availability

The data that support the findings of this study are available upon request.

## Abbreviations

CRC: Colorectal cancer
ChIP-seq: Chromatin immunoprecipitation sequencing
EMT: Epithelial-mesenchymal transition
EV: Empty vector
mtDNA: Mitochondrial DNA
mTOR: Mammalian or mechanistic target of rapamycin
NAC: N-acetyl cysteine
TEM: Transmission electron microscopy
PDX: Patient-derived xenograft
RIP: RNA immunoprecipitation
ROS: Reactive oxygen species
SREBF1: Sterol regulatory element binding transcription factor 1
