# Supplementary material for: SF3B3-regulated mTOR alternative splicing promotes colorectal cancer progression and metastasis
Source: J Exp Clin Cancer Res. 2024 Apr 26;43:126. doi: 10.1186/s13046-024-03053-4 (PMC11047005; doi:10.1186/s13046-024-03053-4)
Supplement: Supplementary file 1 — Additional file 1. Supplementary materials and methods. [file 13046_2024_3053_MOESM1_ESM.docx]

**Reagents and chemicals**

Z-VAD-FMK (Selleck Chemicals, S7023), necrosulfonamide (Selleck Chemicals, S8251), liproxstatin-1 (Selleck Chemicals, S7699), ferrostatin-1 (Selleck Chemicals, S7243), bafilomycin A1 (Selleck Chemicals, S1413), chloroquine (Selleck Chemicals, S6999), and everolimus (Selleck Chemicals, S1120) were obtained from Selleck Chemicals (Houston, TX, USA). N-acetyl cysteine (Sigma-Aldrich, A9165) was bought from Sigma (St. Louis, MO, USA). Rapamycin (SparkJade, 53123-88-9) was obtained from SparkJade Biotechnology (Jinan, China). Curcumin (Solarbio Life Science, IC0610) and palmitate (Solarbio Life Science, H8780) were obtained from Solarbio Life Sicence (Shanghai, China).

**Human samples and ethical statement**

A total of 127 paired human CRC and adjacent noncancerous tissues were obtained from Tianjin Union Medical Center (Tianjin, China). Among these, 98 paired human CRC and adjacent noncancerous tissues were utilized for tissue microarray, while 25 paired tissues were used for qRT-PCR and western blot analysis. Three patient CRC tissues obtained at the time of surgery were used for constructing PDX models. One patient CRC tissue was used for construction of CRC organoids. The study adhered to the recommendations outlined in the Requirements of the Ethical Review System of Biomedical Research Involving Human by Tianjin Union Medical Center Ethics Committee. All subjects were given a written informed consent in accordance with the Declaration of Helsinki.

**Cell culture**

The human CRC cell lines LoVo, HT29, SW480 and other cancer cell lines were obtained from National Infrastructure of Cell Line Resource (Beijing, China). The HEK293T cell line was acquired from Cell Resource Center of Shanghai Institutes for Biological Sciences (Shanghai, China). LoVo, SW480, Hela, MCF7, Huh7, and HEK293T cells were cultured in Dulbecco’s modified Eagle’s medium (DMEM) supplemented with 10% fetal bovine serum (Cell-Box, AUS-01S-02) and 1% penicillin/streptomycin. HT29 cells were maintained in RPMI-1640 medium supplemented with 10% fetal bovine serum and 1% penicillin/streptomycin. All cells were incubated in a humidified incubator at 37°C with 5% CO_2_.

**siRNAs, plasmids and lentivirus**

Specific siRNAs targeting human *SF3B3* and a negative control were designed and synthesized by Gene Pharma (Suzhou, China). Cells were transfected with siRNAs using Lipofectamine RNAiMAX transfection reagent (Thermo Fisher Scientific, 13778150) following the manufacturer’s protocol. RNA and protein were extracted from cells either 48 h or 72 h after transfection. The siRNA sequences are summarized in Supplemental Table S5.

The overexpression plasmid expressing *SF3B3* was constructed by inserting the full-length of human *SF3B3* sequence into the pCDNA3.1 vector (Invitrogen, V790-20). The overexpression plasmid expressing *DHCR24* was constructed by inserting the full-length of human *DHCR24* sequence into the pCDNA3.1 vector. The empty pcDNA3.1 vector was used as a negative control. Full-length *mTOR* overexpression plasmid (+exon8) was purchased from Addgene (http://n2t.net/addgene:26603; RRID: Addgene_26603). Overexpressing plasmid for *mTOR* variant 3 (NM_004958.4) (-exon 8) was constructed by digesting and ligating on the base of the full-length *mTOR*.

The *mTOR* minigene was constructed by PCR amplifying the genomic sequence spanning exons 7 to 9 of human *mTOR* gene and cloning it into the pCDNA3.1 vector. The *DHCR24* minigene was constructed by PCR amplifying the genomic sequence spanning exons 3 to 4 of human *DHCR24* gene and cloning it into the pCDNA3.1 vector. All plasmids were verified by Sanger sequencing, and the cloning primers are listed in Supplemental Table S6.

The knockdown lentivirus plasmids expressing negative control shRNA (shNC) and shRNA against human *SF3B3* (sh*SF3B3*) were cloned into the lentiviral vector pLKO.1 and packaged in HEK293T cells for virus production. Briefly, HEK293T cells were co-transfected with the recombinant plasmids, pMD2.G (Addgene Plasmid 12259), and psPAX2 (Addgene Plasmid 12260) using polyethyenimine (Sigma-Aldrich, 408727) according to the manufacturer’s protocol. After 48 and 72 h post-transfection, the virus was collected and used to infect cells (in the presence of 8 μg/mL polybrene). The stably knockdown cell clones were selected and maintained in culture medium containing 4 μg/mL puromycin. The shRNA sequences are listed in Supplemental Table S7.

**Cell viability and colony formation assays**

For the cell viability assay, cells in suspension were seeded into each well of 96-well plates and cultured for the indicated time. Following incubation with AlamarBlue cell viability reagent (Sigma-Aldrich, R7017) for 4 h, the fluorescence was assessed using Tecan Infinite M200 Microplate Reader with excitation at 540 nm and emission at 590 nm.

For the mechanistic study, cells were treated with various compounds (ZVF, 20 μM; NSA, 10 μM; Lip-1, 1 μM; Fer-1, 1 μM; NAC, 10 mM; Curcumin, 40 μM; CQ, 20 μM; BafA1, 80 nM; Palmitate, 10 μM;) for 24 h or 48 h. The vehicle control was DMSO (the final concentration in the media < 0.1%).

For the colony formation assay, cells in suspension were seeded into each well of 6-well dishes. Cells were cultured for 2 weeks with the medium changed every two days. The colonies were fixed with 4% paraformaldehyde and stained with 0.1% crystal violet (Solarbio Life Science, G1063) for at least 20 min.

**Wound healing, cell-migration and invasion assay**

For the wound healing assay, cells in suspension were seeded into each well of 6-well dishes. Once cells reached to 80% confluence, uniform wounds were created by scraping the cells with a pipette tip. After washing with PBS, wells were refilled with culture medium, and the images were observed and digitally photographed using Nikon Eclipse Ti microscope system (Nikon, Japan).

For the cell migration assay, cells in serum-free medium were seeded into the upper chamber of a Transwell insert pre-coated without (migration assay) or with (invasion assay) Matrigel (BD Biosciences, 356234). The culture medium with 20% FBS was placed into the lower chamber, and cells were further incubated for 24 h. After fixation, the inside cells were carefully scraped off from the upper chamber membrane with cotton swabs, and the cells migrated to the lower membrane surface were stained with 0.1% crystal violet. After washing with PBS, the images of migrated and invaded cells were digitally photographed using Nikon Eclipse Ti microscope system (Nikon, Japan).

**RNA isolation and PCR assay**

Total RNA was isolated using RNAiso Plus (Takara, 9109). RNA was reverse-transcribed into cDNA using a HiScript Q RT SuperMix (Vazyme, R223-01). qRT-PCR was performed on ABI QuantStudio 6 Flex Real-Time PCR system (Applied Biosystems, CA, USA) using Ultra SYBR Mixture (CWBIO, CW2601M) with specific primers. The expression data were analyzed using 2^-∆∆Ct^ method to quantify the fold change in mRNA levels. *GAPDH* and *ACTB* were used as housekeeping genes. For PCR, the amplified products were visualized by separation on 1-3% agarose gels. Primer sequences are provided in Supplemental Table S8.

**Protein extraction and Western blot analysis**

Total protein was extracted using RIPA lysis buffer containing protease/phosphatase inhibitors. The protein concentrations were determined using a BCA protein assay kit (CWBIO, CW0014S). Equal amounts of protein were separated by electrophoresis on 8-15% SDS-PAGEs and then transferred onto PVDF membranes. Following blocking, membranes with the target proteins were probed with primary antibodies overnight at 4°C. The membranes were then washed and incubated with HRP-conjugated secondary antibodies for 1 h at room temperature (antibody list is shown in Supplemental Table S9). The signal bands were viewed using the Amersham Imager 600 (GE Healthcare, MA, USA). The relative protein levels were quantified using Image J software by comparing the grayscale of the western blot bands.

**Immunohistochemistry (IHC) staining**

IHC staining was performed according to standard protocols. Briefly, the specimens were deparaffinized in xylene and rehydrated in a graded series of ethanol. Following antigen retrieval performed in citrate unmasking solution by heating in a microwave, the specimens were incubated with 3% hydrogen peroxide for 10 min. The sections were blocked with 10% normal goat serum for 30 min at room temperature and incubated with the primary antibody overnight at 4°C (antibody list is shown in Supplemental Table S9). The next day, sections were washed with PBS and incubated with secondary antibody for 2 h. Sections were stained with 3, 3′-Diaminobenzidine (DAB) and nuclear counterstaining was performed using hematoxylin. The sections were digitized using Aperio scanners (Aperio CT6, LEICA, Germany). IHC staining was quantified using the immunoreactive score (IRS) system by Image J software.

The staining extent was scored on a scale of 0−4: (0) if 0% of tumor cells were positive, (1) if <10% were positive, (2) if 11%-50% were positive, (3) if 51−80% were positive, and (4) if >80% were positive. The staining intensity was scored at 0-3 (3 is the highest positivity). The final IHC score was generated by multiplying the score of staining extent with the score of staining intensity (The maximum score is 12).

**Immunofluorescence staining**

Cells were seeded on sterile coverslips in a 24-well plate and then treated as indicated. After washed with PBS three times, cells were fixed with 4% paraformaldehyde for 15 min and then permeabilized with PBS containing 0.3% Triton-X100 (Solarbio life sciences, T8200) for 5 min. Cells were blocked with PBS containing 2% BSA for 1 h and then incubated with anti-LC3 primary antibody solution at 4°C overnight. The next day, cells were washed with PBS containing 0.1% Tween-20 three times and incubated with a secondary immunofluorescent antibody for 1 h at room temperature. Cells were then stained with DAPI (Solarbio life sciences, C0065) for 5 min to visualize nuclei. Cells were mounted to slices, and images were captured using Leika confocal microscopy (Leika SP8, Mannheim, Germany).

**Apoptosis assay**

Cells were harvested, washed twice using cold PBS, and then re-suspended in 100 μL of 1x binding buffer. Cell were incubated in binding buffer containing 5 μL of Annexin V-FITC and 5 μL of propidium iodide (Annexin V-FITC/PI Apoptosis Detection Kit, Meilun Biotechnology, MA0220) in the dark for 15 min at room temperature. Cell apoptosis ratios were analyzed by flow cytometry using BD FACSAria III (BD Biosciences, NJ, USA). All samples were analyzed by the FlowJo software (BD Biosciences, NJ, USA).

**TUNEL staining**

Cells were seeded on sterile coverslips. After treatment, cells were fixed in 4% paraformaldehyde. TdT reaction mix was added to coverslips and incubated for 1 h at 37°C in a humidified chamber using the TUNEL Apoptosis Assay Kit (Beyotime Biotechnology, C1088). Cells were stained with DAPI to visualize nuclei. Fluorescence images were captured using Nikon Eclipse Ti microscope system (Nikon, Japan).

**mCherry-GFP-LC3 transfection and autophagy flux detection**

LoVo and HT29 cells transfected with Lenti-mCherry-GFP-LC3 (Beyotime, C3002) were seeded on sterile coverslips in a 24-well plate. After treatment, images were captured using Leika confocal microscopy (Leika SP8, Mannheim, Germany).

**Mito-tracker staining and measurement of mtDNA copy number**

Cells were stained with Mito-Tracker Green (Beyotime, C1048) reagent for 30 min at 37 °C, and the nuclei were stained with Hoechst33258 (Beyotime, C1027) for 5 min. The images were captured using Leika confocal microscopy (Leika SP8, Mannheim, Germany).

Total genomic DNA was extracted from cells using the Genomic DNA miniprep kit (Genstone biotech, TD468) according to the manufacturer’s instructions. DNA concentration was measured by NanoDrop (MIULAB ND-100, Zhejiang, China). Mitochondrial DNA was quantified using qRT-PCR. Nuclear *β-globin* gene was used as the internal control. Primer sequences are listed in Supplemental Table S8.

**Transmission electron microscopy (TEM)**

Cells were fixed with 2.5% glutaraldehyde overnight at 4°C, and washed with PBS. Cell pellets were pooled and fixed with 1% OsO4 buffer and 0.5% potassium ferricyanide for 1.5 h at 4°C. After that, cells were dehydrated in a graded series of ethanol solutions and embedded in straight resin. Ultra-thin sections were cut on copper grids, the blocks were double-stained with 1% uranyl acetate and 0.2% lead citrate. Sections were visualized by transmission electron microscope (Hitachi-7800, Japan).

**Determination of reactive oxygen species (ROS) production**

Cells were seeded in a 24-well plate and treated as indicated. Cells were then stained with ROS indicator DCFH-DA (10 μM) in the dark at 37°C for 30 min. Afterward, the images were digitally photographed using Nikon Eclipse Ti microscope system (Nikon, Japan). Cells were harvested with PBS and the ROS level was assessed with excitation at 488 nm and emission at 525 nm using Tecan Infinite M200 Microplate Reader.

**Triglyceride (TG) measurement**

Cells were collected and resuspended. The cell pellets were added to PBS containing 1% triton-100, and were lysed by sonication on ice. TG in the lysate was measured using a TG assay kit (Nanjing Jiancheng Bioengineering Institution, A110-1-1) according to the manufacturer’s protocol.

**Nile red staining**

Cells were seeded on sterile coverslips in a 24-well plate. After PBS wash, cells were fixed with 4% paraformaldehyde for 20 min and stained with 0.01 mM Nile red (Yuanye Biotechnology, S19279) in the dark for 15 min. Cells were stained with DAPI for 5 min to visualize nuclei. After PBS wash, fluorescence images were captured using Nikon Eclipse Ti microscope system (Nikon, Japan). The fluorescence was determined using Tecan Infinite M200 Microplate at 543 nm excitation and 598 nm emission.

**RNA sequencing**

Total RNA from LoVo cells transiently transfected with either *SF3B3* siRNAs or negative control siRNAs (siNC) was isolated using RNAiso Plus according to the manufacturer’s protocol. Next-generation sequencing library preparations and NOVAseq6000 sequencing were performed by GENEWIZ Corporation (Jiangsu, China). Differential expression genes were determined by counting expression with HTSeq and testing with edgeR and DESeq. Only those genes with |log2(FC)| of > 1 and an adjusted *P* value of <0.05 were considered differentially expressed. Differentially alternative splicing patterns from RNA-Seq were quantified in *SF3B3*-knockdown samples compared to negative controls using the rMATS tool (v 4.0.1). Additionally, functional enrichment pathway analysis of differential AS events was performed using KOBAS 3.0 database.

**RNA immunoprecipitation (RIP)**

RIP assay was conducted using RNA Immunoprecipitation (RIP) Kit (BersinBio, Bes5101) according to the manufacturer’s protocol. Briefly, LoVo cells were lysed in polysome lysis buffer. The RNA-protein complexes were divided into anti-SF3B3 (Sigma-Aldrich, HPA042986), anti-IgG and input samples, respectively, which were incubated with the respective antibody overnight at 4°C, followed with balanced protein A/G beads at 4°C for 1 h. After washing, the bead-bound complexes were digested with proteinase K. The immunoprecipitated RNA was extracted, purified, reverse-transcribed into cDNA, and subjected to PCR analysis and agarose gel electrophoresis.

**Targeted lipidomics**

LoVo cells transiently transfected with either *SF3B3* siRNAs or negative control siRNAs were washed twice with cold PBS and harvested in 2 mL centrifuge tubes. Subsequently, homogenization was carried out with 1 mL of chloroform methanol (2:1, v/v). After centrifugation at 12000g for 10 min at 4℃, the supernatant was collected and 2 mL of sulfuric acid-methanol solution was added for esterification. Next, 1 mL of n-hexane was added to the mixture, followed by centrifugation at 3500g for 10 min at 4°C. The supernatant was obtained and internal standards were added. Lipidomic analysis was performed using Thermo Trace 1300 coupled to ISQ 7000 mass spectrometry (Thermo Scientific) at Shanghai Bioprofile Technology Co., Ltd. (Shanghai, China).

**Luciferase reporter assay**

The pGL4 firefly luciferase reporters (PGL4-SREBF1a and PGL4-SREBF1c) containing *SREBF1a* (-1037 to +10) or *SREBF1c* (-1003 to -252) promoter regions were constructed by PCR amplifying the genomic sequence of the human *SREBF1* gene. The *SREBF1c* promoter region (-251 to +1) was skipped due to the GC-rich sequence. The constructs were confirmed by Sanger sequencing. HEK293T cells were transfected with shSF3B3#1 lentivirus (shNC vs shSF3B3) or overexpressing plasmids (empty vector, EV; SF3B3 overexpressing plasmid, SF3B3) for 48 h, followed by transfection with pGL4-SREBF1a and pGL4-SREBF1c together with the pRL-TK plasmid containing the Renilla luciferase reporter gene for 24 h. Subsequently, luciferase activity was detected with a Dual-Luciferase Reporter Assay System (Promega Inc, E1910) using Tecan Infinite M200 Microplate Reader. Data were normalized by the ratio of Firefly to Renilla luminescence.

**Chromatin immunoprecipitation (ChIP)**

ChIP assay was performed using a ChIP Kit (Abcam, ab500) according to the manufacturer’s protocol. Briefly, LoVo cells were crosslinked with 1% formalin solution for 10 min and quenched with 10 x glycine. Cell lysates were harvested and sonicated to produce chromatin fragments. Immunoprecipitation was carried out by adding anti-H3K27ac and IgG antibodies. The immunoprecipitated DNAs fragments were purified and analyzed by PCR. The primers and antibodies are listed in Supplemental Table S8-9.

**Bioinformatics analysis**

TCGA datasets were downloaded from the University of California Santa Cruz Xena dataset (http://xena.ucsc.edu/). The Gene Expression Profiling Interactive Analysis (GEPIA) website (http://gepia2.cancer-pku.cn/) was utilized to explore gene expression based on TCGA-COAD, TCGA-READ, and GTEx datasets. The cBioPortal website (https://www.cbioportal.org/) was utilized to analyze the KEGG pathway obtained from the colorectal adenocarcinoma datasets in the Pan-Cancer Atlas of TCGA. The pathway diagrams were plotted by an online platform (https://www.bioinformatics.com.cn). CRC datasets (GSE223119, GSE142279, GSE 166254, GSE83968, GSE96069, GSE71510) were available on GEO (http://www.ncbi.nlm.nih.gov/geo). Cistrome DB Toolkit (http://dbtoolkit.cistrome.org) was used to analyze the potential epigenetic factors and UCSC Genome Browser (http://genome.ucsc.edu) was used for visualizing genomic data.

**Animal studies**

BALB/C nude mice (male and female, 4-5 weeks of age) were obtained from Beijing Vital River Laboratory Animal Technology Co., Ltd. (Beijing, China). NSG mice (female, 6-8 weeks of age) were obtained from Gem Pharmatech (Jiangsu, China). The protocol for mouse housing and usage was approved by the Laboratory Animal Ethics Committee of Tianjin Haihe hospital (Tianjin, China).

For LoVo cell xenograft models, 1 × 10^7^ stably transfected cells suspended in 0.2 mL of PBS were subcutaneously injected into flank region of each BALB/C nude mouse. The mice were then monitored for tumor size using caliper measurements and overall health every three days. Tumor growth was calculated based on two perpendicular measurements using the following formula: V_volume_ (mm^3^) = 0.5 × L_length_× W_width_^2^. Mice were sacrificed at the experimental endpoint, and subcutaneous tumors were removed, weighed, and resected for further analysis.

For patient-derived xenograft (PDX) models, the CRC tumor tissues were cut into the 2-3 mm^3^ pieces and transplanted into the flank region of BALB/c nude mice using a TROCHAR. When the tumor volume reached 1–2 cm^3^, the xenografted tumors were harvested, cut into pieces, and further implanted into BALB/c nude mice. When the xenografted tumors reached approximately 50 mm^3^, mice were randomly divided into two groups for lentivirus treatment (shNC lentivirus and sh*SF3B3* lentivirus). Lentiviruses produced in HEK293T cells were concentrated by incubation with PEG8000 (Sigma-Aldrich, 89510) for 4 h and centrifugation at 3000g for 30 min. Mice in different groups received the corresponding viruses via intratumor injection at multiple spots for a total of four times. Tumor weights and volume were recorded. At the end of the study, the tumors were harvested for further analysis.

To explore the synergistic effect of *SF3B3* shRNAs and mTOR inhibitors, PDX models of nude mice were intratumorally injected viruses at multiple spots for a total of four times followed by oral gavage of vehicle (40% PEG400, 5% Tween-80 and 5% DMSO) and everolimus (5mg/kg, every two days for 3 weeks).

For lung metastasis models, a tail vein injection experiment in nude mice was performed. Nude mice were randomly divided into two groups, with each animal injected with 5× 10^6^ stably knockdown LoVo or HT29 cells in 200 uL of PBS via tail vein. Mice were sacrificed eight weeks post-injection, and lung tissues were collected for further analysis.

For orthotopic liver metastasis models, procedures were carried out following the previously published methods[1,2]. Mice were anesthetized with isoflurane, and the abdomens were sterilized with iodine and alcohol swabs. Afterward, 5×10^6^ stably knockdown LoVo cells in 50 uL of PBS were injected into mouse cecum wall. Mice were maintained in a sterile environment. Mice were sacrificed seven weeks after surgery. Liver and cecum tissues were collected for further analysis. Schematic illustration of model in the manuscript were drawn in Figdraw.

**H&E staining**

Tissues were collected, fixed in 4% formalin, and then embedded in paraffin. The paraffin tissues were cut into 5 μm slices and were stained with hematoxylin and eosin (Solarbio Life Science, G1120) following standard procedures. The sections were digitized using Aperio scanners (Aperio CT6, LEICA, Germany).

**Patient-derived CRC organoids and treatment**

Organoids were cultured in organoid culture medium (DMEM/F12 medium with 10 mM HEPES, 2 mM Glutamax, B27 supplement, 1× N2 supplement, 50 ng/mL m-EGF, 1 mM N-acetylcysteine, and penicillin-streptomycin). To achieve *SF3B3* knockdown, digested organoids were infected with lentivirus for 6 h at 37°C, followed by reconstitution in Matrigel in 24-well plate. After culturing for 7 days, organoids were further cultured in medium containing 40 μM everolimus. The images were captured using Nikon Eclipse Ti microscope system (Nikon, Japan).

**Statistical Analysis**

All data are expressed as the mean ± standard deviation (SD) values. Statistical comparisons were determined Student's t test for two groups or one-way analysis of variance (ANOVA). The survival curves were generated using the Kaplan-Meier method. Survival analysis was conducted using the log-rank test. The correlation between two groups was analyzed using Pearson’s test. Statistical significance was considered with a *P* value < 0.05. All statistical analyses were performed using GraphPad Prism software version 8.0 (Graph Pad Software Inc., La Jolla, CA).

**Reference**

1. Céspedes MV, Espina C, García-Cabezas MA, Trias M, Boluda A, Gómez del Pulgar MT, et al. Orthotopic microinjection of human colon cancer cells in nude mice induces tumor foci in all clinically relevant metastatic sites. Am J Pathol. 2007;170:1077-85.

2. Zhang L, Zhu Z, Yan H, Wang W, Wu Z, Zhang F, et al. Creatine promotes cancer metastasis through activation of Smad2/3. Cell Metab. 2021;33:1111-1123.e4.
