# Supplementary material for: SF3B3-regulated mTOR alternative splicing promotes colorectal cancer progression and metastasis
Source: J Exp Clin Cancer Res. 2024 Apr 26;43:126. doi: 10.1186/s13046-024-03053-4 (PMC11047005; doi:10.1186/s13046-024-03053-4)
Supplement: Supplementary file 3 — Additional file 3. Supplementary figures and tables. Figure S1. SF3B3 is upregulated in human CRC and regulated by H3K27ac. (A) SF3B7 transcript levels in normal and CRC tissues. TCGA-COAD and TCGA-READ datasets (normal, n=51; cancer, n=383) were obtained from Xena (http://xena.ucsc.edu/). Data are shown as mean ± SD. (B) mRNA levels of SF3b family members in CRC tissues from TCGA-COAD and TCGA-READ datasets in cBioPortal (https://www.cbioportal.org/). (C) mRNA levels of SF3b family members in normal and CRC tissues after integrating the data of normal colon tissues from GTEx datasets with TCGA datasets. Analysis was performed by GEPIA2 (http://gepia2.cancer-pku.cn/). (D) KEGG pathway analysis of SF3B3 positively and negatively co-expressed genes that were identified using cBioPortal (https://www.cbioportal.org/). (E) Correlation analysis between SF3B3 and MKI67 mRNA levels in TCGA datasets from Xena (http://xena.ucsc.edu/). (F) Potential epigenetic factors for SF3B3 gene using a parameter of 1-kb regulatory potential decay in Cistrome DB Toolkit (http://dbtoolkit.cistrome.org). (G) Visualization of H3K27ac enrichment in the promoter region of SF3B3 gene using UCSC Genome Browser (http://genome.ucsc.edu/). (H) Genome browser view of H3K27ac occupancy in SF3B3 promoter region. ChIP-Seq data from three CRC cell lines (GSE83968, GSE96069, and GSE71510) were retrieved from GEO datasets (http://www.ncbi.nlm.nih.gov/geo). (I) SF3B3 mRNA levels in LoVo and HT29 cells treated with 40 μM curcumin for 24 h. FOXM1 was used as positive control for H3K27ac target. Data are shown as mean ± SD. Figure S2. SF3B3 promotes proliferation and metastasis in vitro. (A) qRT-PCR quantification of SF3B3 mRNA expression as well as representative western blots of SF3B3 in 7 CRC cell lines and normal human colon mucosal epithelial cell line (NCM460). (B) qRT-PCR analysis of SF3B3 mRNA as well as representative western blots of SF3B3. LoVo and HT29 cells were transfected with siRNAs and collect [file 13046_2024_3053_MOESM3_ESM.docx]

**Supplemental Figures**

**
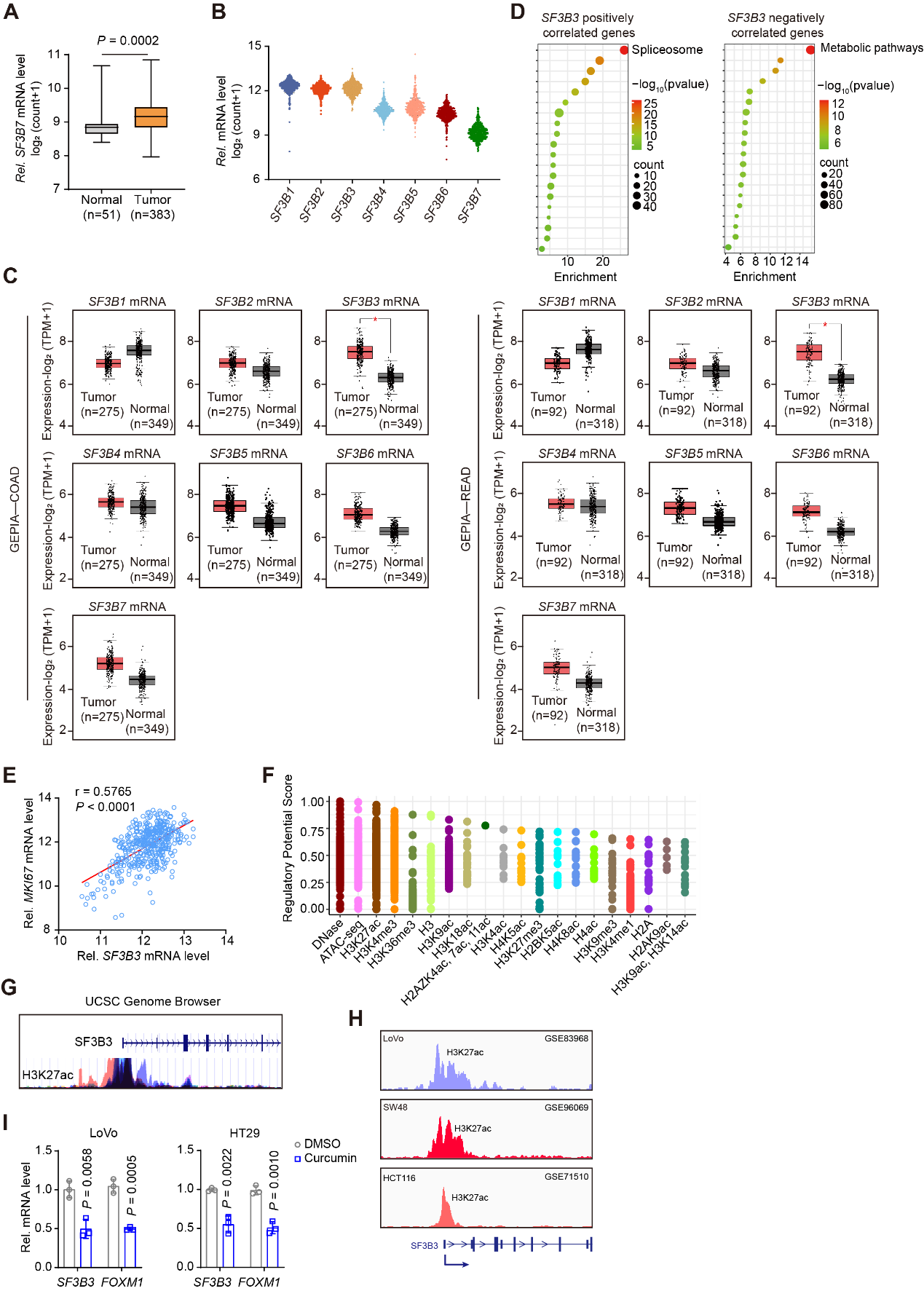
**

**Figure S1. *SF3B3* is upregulated in human CRC and regulated by H3K27ac.** (**A**) *SF3B7* transcript levels in normal and CRC tissues. TCGA-COAD and TCGA-READ datasets (normal, n=51; cancer, n=383) were obtained from Xena (http://xena.ucsc.edu/). Data are shown as mean ± SD. (**B**) mRNA levels of SF3B family members in CRC tissues from TCGA-COAD and TCGA-READ datasets in cBioPortal (https://www.cbioportal.org/). (**C**) mRNA levels of SF3B family members in normal and CRC tissues after integrating the data of normal colon tissues from GTEx datasets with TCGA datasets. Analysis was performed by GEPIA2 (http://gepia2.cancer-pku.cn/). (**D**) KEGG pathway analysis of *SF3B3* positively and negatively co-expressed genes that were identified using cBioPortal (https://www.cbioportal.org/). (**E**) Correlation analysis between *SF3B3* and *MKI67* mRNA levels in TCGA datasets from Xena (http://xena.ucsc.edu/). (**F**) Potential epigenetic factors for *SF3B3* gene using a parameter of 1-kb regulatory potential decay in Cistrome DB Toolkit (http://dbtoolkit.cistrome.org). (**G**) Visualization of H3K27ac enrichment in the promoter region of *SF3B3* gene using UCSC Genome Browser (http://genome.ucsc.edu/). (**H**) Genome browser view of H3K27ac occupancy in *SF3B3* promoter region. ChIP-Seq data from three CRC cell lines (GSE83968, GSE96069, and GSE71510) were retrieved from GEO datasets (http://www.ncbi.nlm.nih.gov/geo). (**I**) *SF3B3* mRNA levels in LoVo and HT29 cells treated with 40 μM curcumin for 24 h. *FOXM1* was used as positive control for H3K27ac target. Data are shown as mean ± SD.

**
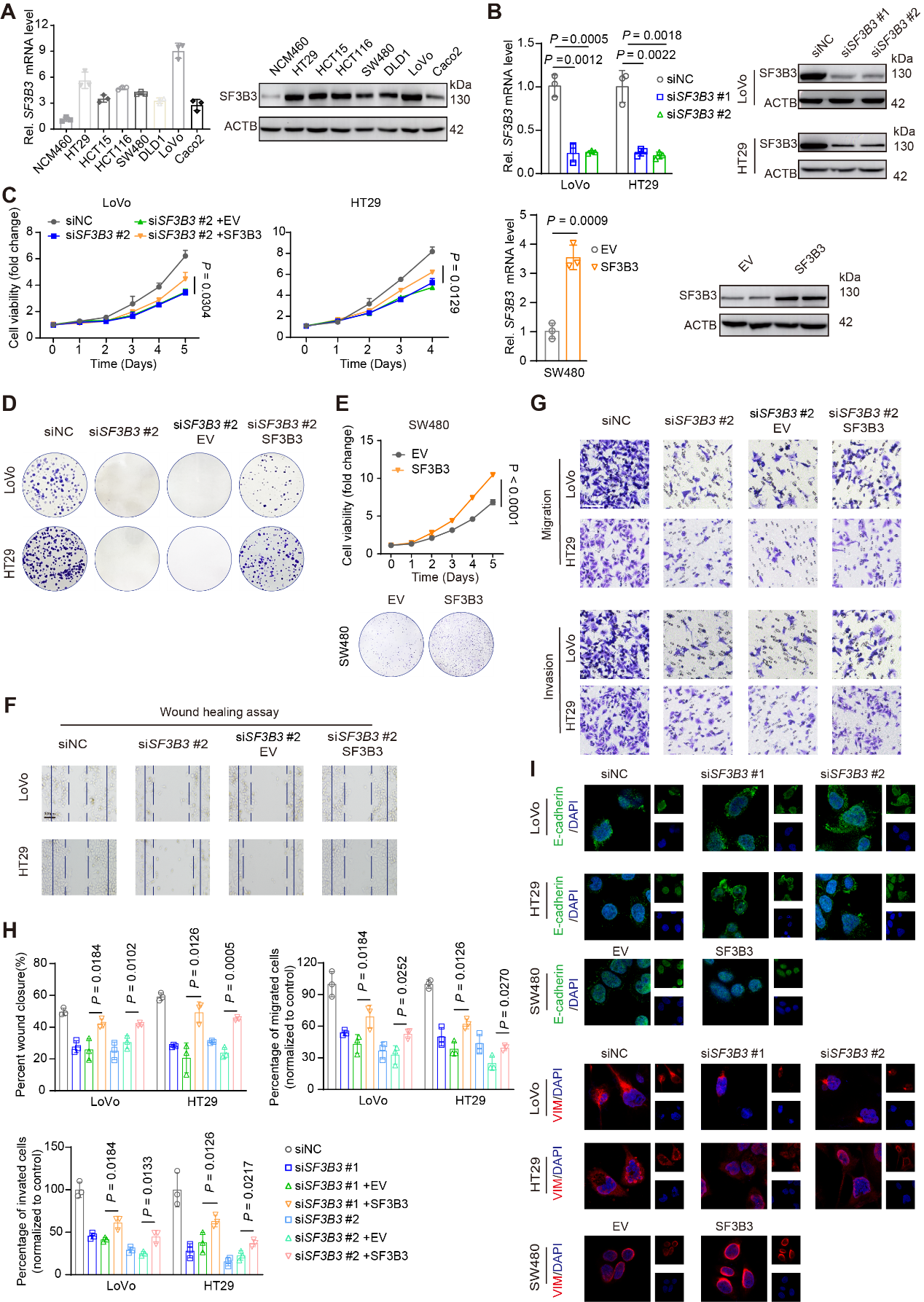
**

**Figure S2. SF3B3 promotes proliferation and metastasis in *vitro*.** (**A**) qRT-PCR quantification of *SF3B3* mRNA expression as well as representative western blots of SF3B3 in 7 CRC cell lines and normal human colon mucosal epithelial cell line (NCM460). (**B**) qRT-PCR analysis of *SF3B3* mRNA as well as representative western blots of SF3B3. LoVo and HT29 cells were transfected with siRNAs and collected at 48 h for mRNA detection or at 72 h for protein detection. SW480 cells were transfected with *SF3B3*-overexpressing plasmids for 72 h. (**C**) Growth curves and (**D**) colony formation of LoVo and HT29 cells after *SF3B3* knockdown by siRNAs (siNC vs si*SF3B3*#2), or after *SF3B3* knockdown by siRNAs for 12 h followed with re-expression of *SF3B3* (si*SF3B3*#2+EV vs si*SF3B3*#2+SF3B3) (empty vector, EV; SF3B3 overexpressing plasmid, SF3B3). (**E**) Growth curves and colony formation of SW480 cells after transfection with *SF3B3* overexpressing plasmids. (**F**) Wound healing assays and (**G**) Transwell assays were used to measure cell migration and invasion abilities. LoVo and HT29 cells were treated with siRNAs (siNC vs si*SF3B3*#2) to knockdown *SF3B3*. For *SF3B3* re-expression study, cells were treated with siRNAs (siNC vs si*SF3B3*#2) for 12 h, followed by transfection with EV or *SF3B3* overexpressing plasmids (si*SF3B3*#2+EV vs si*SF3B3*#2+SF3B3). Scale bars, 100 μm. (**H**) Statistical analysis of Wound healing and Transwell assay results. Data are shown as mean ± SD. (**I**) Representative immunofluorescence staining images of E-cadherin (green), VIM (red) and DAPI (blue) in CRC cells using a confocal microscope with 100× oil immersion lens.


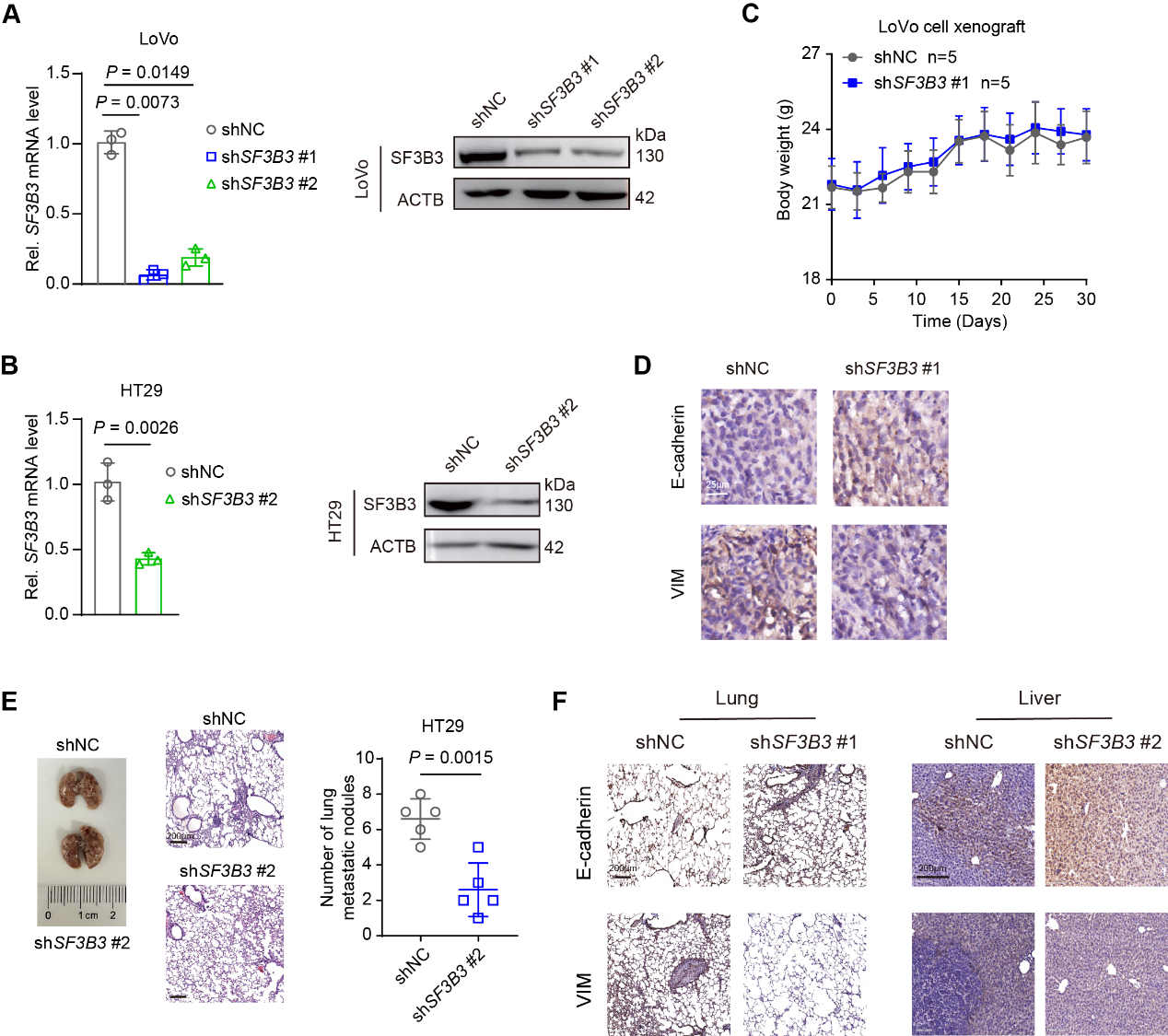


**Figure S3. Silencing of *SF3B3* impedes CRC proliferation and metastasis in *vivo*.** qRT-PCR and western blot analyses of stably *SF3B3*-knockdown LoVo (**A**) and HT29 (**B**) cells. LoVo cells were infected with sh*SF3B3*#1 or sh*SF3B3*#2 lentivirus, whereas HT29 cells were infected with sh*SF3B3*#2 lentivirus. (**C**) Body weights of LoVo cell xenograft nude mice. The stably *SF3B3*-knockdown LoVo cells (LoVo-sh*SF3B3*#1) were subcutaneously injected into flank region of nude mice. (**D**) Representative IHC images of E-cadherin and VIM proteins in LoVo-shNC and LoVo-sh*SF3B3*#1 xenografts. Scale bars, 25 μm. (**E**) Representative images of lung, H&E staining for lung tissues, and statistical analysis of metastatic nodules in lung (n=5/group). Control and *SF3B3*-knockdown HT29 cells (sh*SF3B3*#2) were injected into nude mice via the tail vein. Scale bars, 200 μm. Data are shown as mean ± SD. (**F**) Representative IHC images of E-cadherin and VIM proteins in metastasis lung and liver tissues. Scale bars, 200 μm.

**
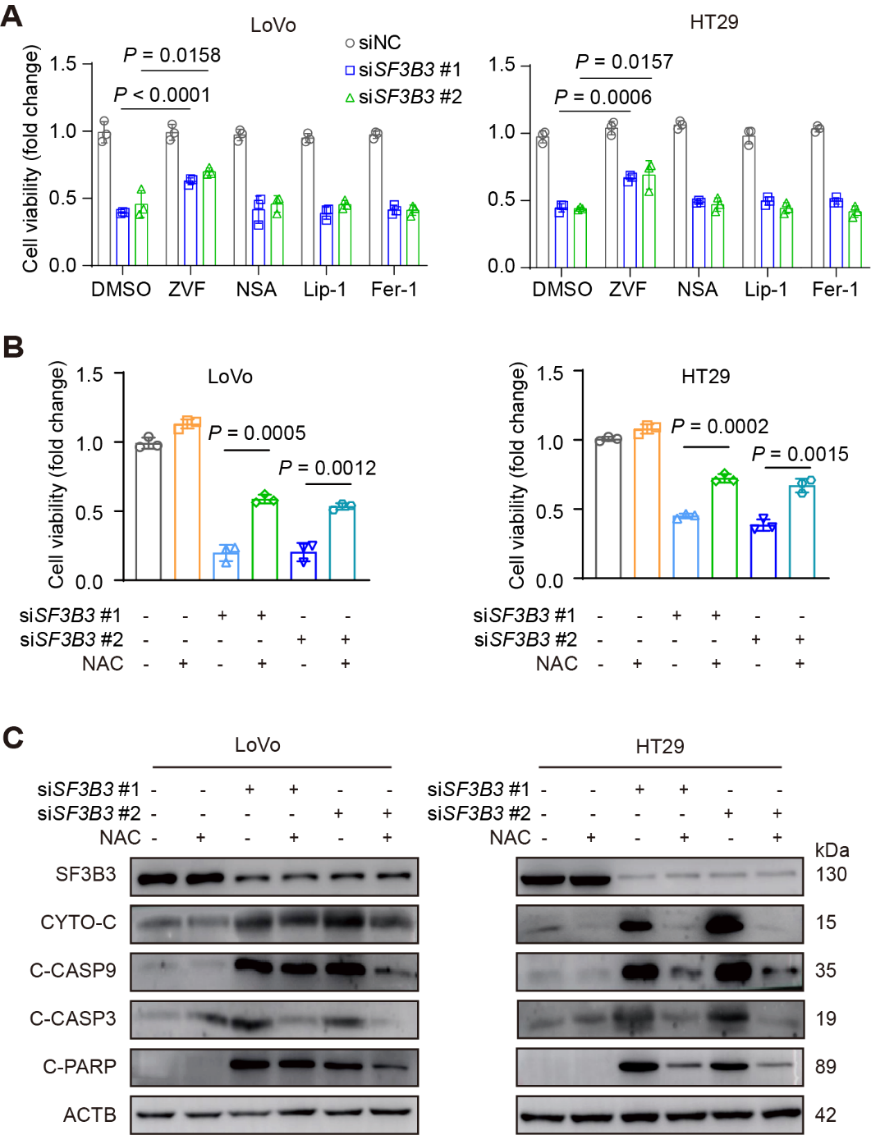
**

**Figure S4. SF3B3 regulates mitochondria-mediated apoptosis in CRC cells.** (**A**) Cell viability of *SF3B3*-knockdown CRC cells co-treated with apoptosis-, necrosis- or ferroptosis-inhibitors. LoVo and HT29 cells were transfected with siRNAs for 24 h, followed by treatment with 20 μM ZVF (Z-VAD-FMK), 10μM NSA (necrosulfonamide), 1μM Lip-1 (liproxstatin-1) or 1μM Fer-1 (ferrostatin-1) for 48 h. (**B**) Cell viability of *SF3B3*-knockdown CRC cells co-treated with ROS inhibitor. LoVo and HT29 cells were transfected with siRNAs for 24 h, followed by treatment with 10 mM NAC (N-acetylcysteine) for 48 h. (**C**) Representative western blots of apoptosis-related proteins. LoVo and HT29 cells were transfected with siRNAs for 24 h, followed by 10 mM NAC for 48 h. Data are shown as mean ± SD.


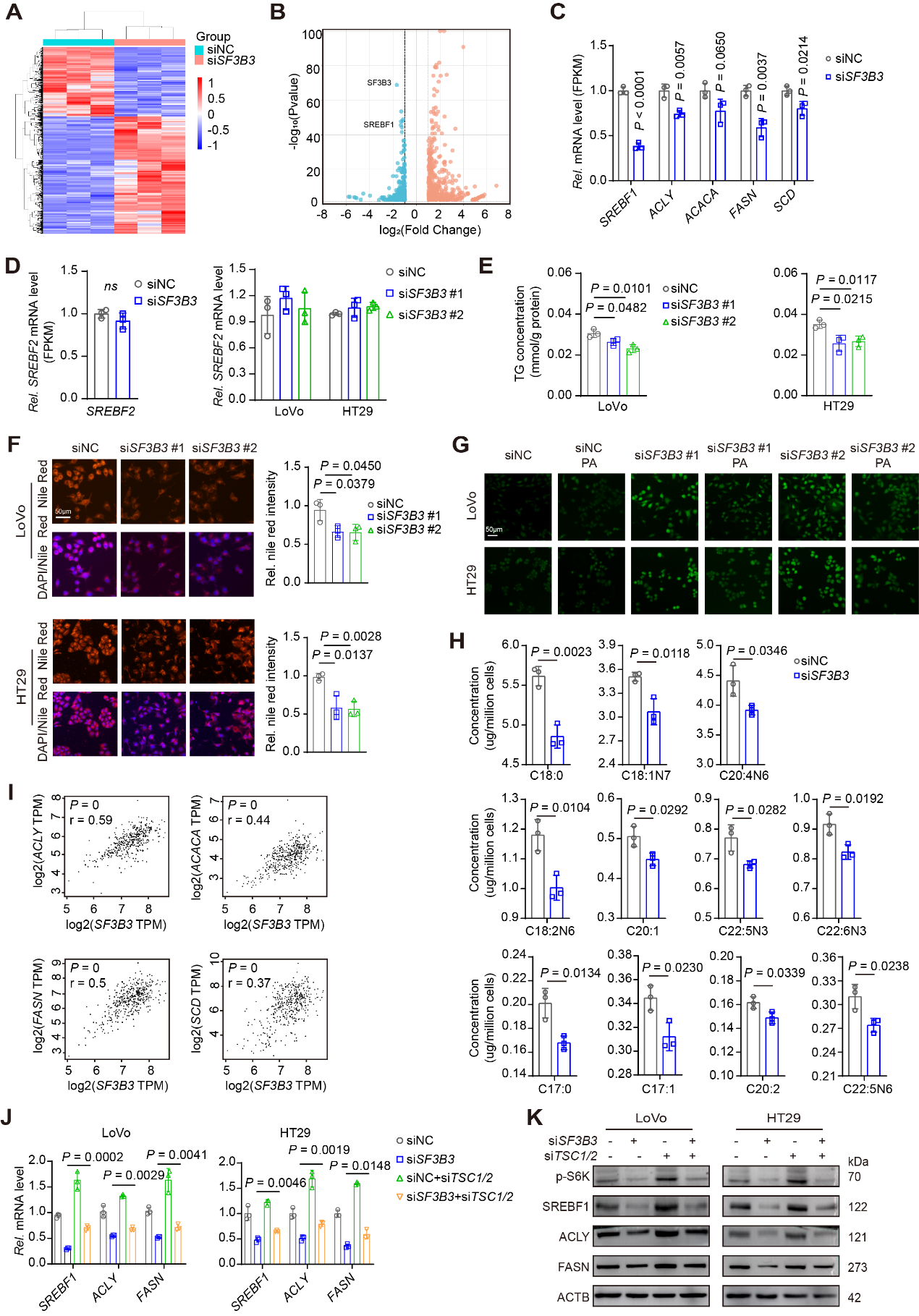


**Figure S5. SF3B3 regulates lipogenesis in CRC cells via SREBF1-FASN signaling.** (**A**) Heatmap and (**B**) volcano plot displaying the significantly differential expression genes from RNA-seq of LoVo cells after transfection with siRNAs (mixture of si*SF3B3*#1 and si*SF3B3*#2) for 48 h. (**C**) Transcript levels of lipogenesis-related genes from RNA-seq of control and *SF3B3*-knockdown LoVo cells. (**D**) Transcript levels of *SREBF2* in RNA-seq analysis of *SF3B3*-knockdown LoVo cells, as well as qRT-PCR analysis of *SREBF2* mRNA in CRC cells after transfection with siRNAs for 48 h. (**E**) Triglyceride levels in *SF3B3*-knockdown CRC cells after transfection with siRNAs for 72 h. (**F**) Nile red staining (Red) images and analysis of *SF3B3*-knockdown CRC cells after transfection with siRNAs for 72 h. Nuclei (blue) were stained with DAPI. Scale bars, 50 μm. (**G**) Representative fluorescence images of ROS (green) staining. CRC cells were transfected with siRNAs for 24 h, followed by treatment with 10 μM palmitate for 48 h. Scale bars, 50 μm. (**H**) Concentrations of individual fatty acids in LoVo cells after transfection with siRNAs (mixture of si*SF3B3*#1 and si*SF3B3*#2) for 72 h. The data are calculated based on lipidomic study. (**I**) Correlation analysis of the mRNA expression between *SF3B3* and lipogenesis-related genes based on TCGA-COAD and TCGA-READ datasets using GEPIA2. (**J**) qRT-PCR quantification of the mRNA expression of *SREBF1* and its target genes. (**K**) Representative western blots of p-S6K, SREBF1, ACLY, and FASN. LoVo and HT29 cells were transfected with siRNAs (mixture of si*SF3B3*#1 and si*SF3B3*#2) for 12 h, followed by transfection with combined si*TSC1* and si*TSC2* for 60 h. Data are shown as mean ± SD.


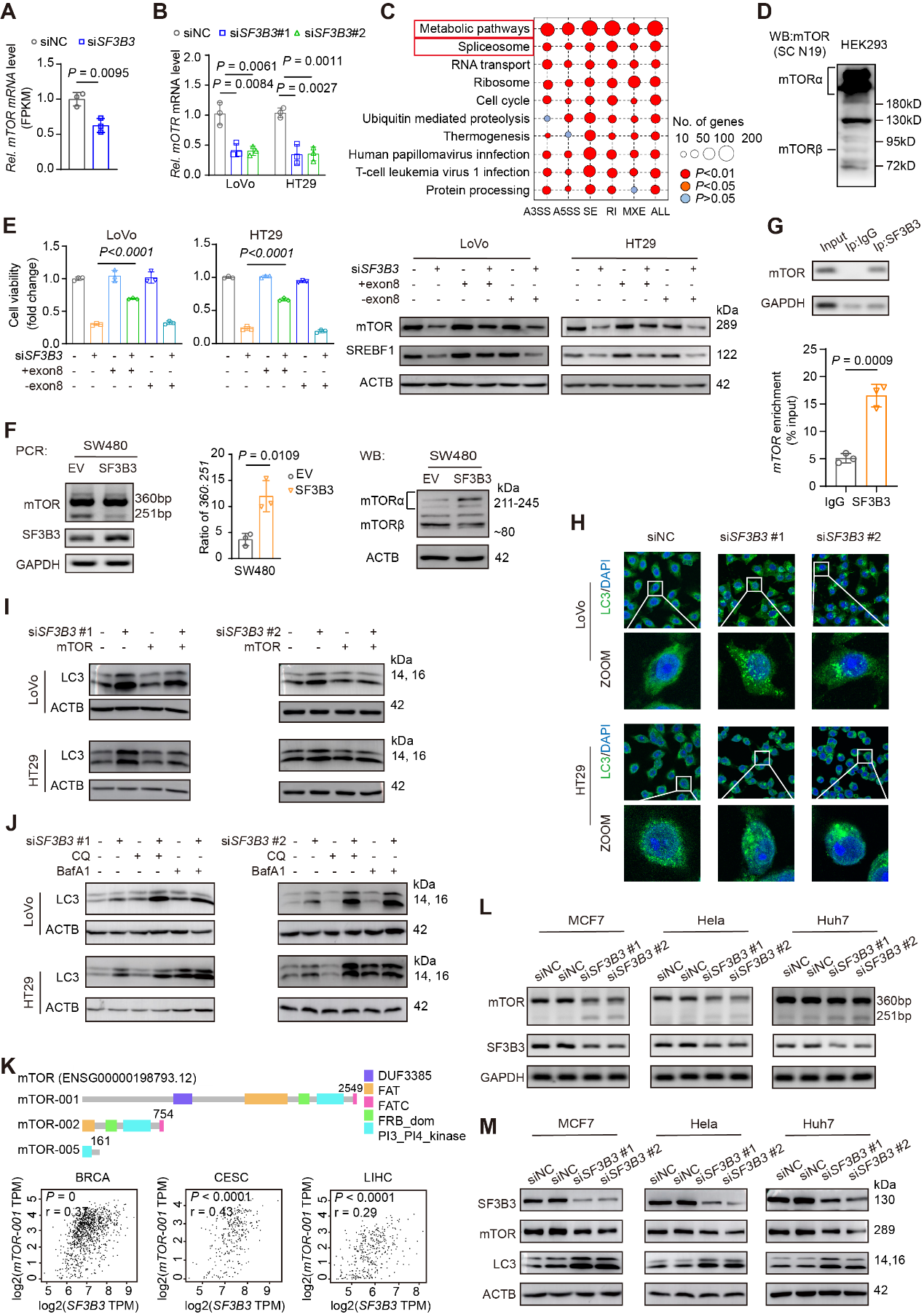


**Figure S6.** **SF3B3 regulates mTOR splicing and autophagy.** (**A**) mRNA levels of *mTOR* in *SF3B3*-knockdown LoVo cells from RNA-seq data. (**B**) mRNA expression of *mTOR* was quantified by qRT-PCR in CRC cells after transfection with siRNAs for 48 h. (**C**) KEGG pathway enrichment analysis of *SF3B3*-regualted AS events from RNA-seq data. Differentially alternative splicing patterns were determined using the rMATS tool. (**D**) Representative western blots of mTORα and mTORβ in the protein extracts of HEK293T cells using previous reported N-terminal mTOR antibody (sc-517464). (**E**) Cell viability and representative western blots of mTOR and SREBF1 of *SF3B3*-knockdown CRC cells after *mTOR* (+exon8 or -exon8) overexpression. LoVo and HT29 cells were transfected with si*SF3B3*#1 for 12 h, followed by transfection with full-length *mTOR* (+exon 8)- and *mTOR*-*variant 3* (-exon 8)-overexpressing plasmids for 60 h. (**F**) Exon 8 skipping of *mTOR* was examined by 3% agarose gel electrophoresis of PCR products in SW480 cells. Representative western blots of mTORα and mTORβ in SW480 cells using the mTOR antibody (sc-517464). SW480 cells were transfected with *SF3B3*-overexpressing plasmids for 72 h. Data are shown as mean ± SD. (**G**) RNA-IP analysis of the enrichment of *mTOR* in SF3B3 protein-antibody-beads system in LoVo cells. (**H**) Representative immunofluorescence images of LC3 (green) and DAPI (blue) in *SF3B3*-knockdown CRC cells using a confocal microscope with 63× oil immersion lens. LoVo and HT29 cells were transfected with siRNAs for 72 h. (**I**) Representative western blots of LC3B in *SF3B3*-knockdown CRC cells after forced expression of *mTOR*. LoVo and HT29 cells were transfected with siRNAs for 12 h, followed by transfection with *mTOR*-overexpressing plasmids for 60 h. (**J**) Representative western blots of LC3 in *SF3B3*-knockdown CRC cells after treatment with autophagy inhibitors. LoVo and HT29 cells were transfected with siRNAs for 24 h, followed by treatment with 20 μM CQ or 80 nM BafA1 for 48 h. (**K**) mTOR isoform structures identified in TCGA datasets (illustrated by GEPIA2). Correlation analysis between the *SF3B3* mRNA levels and full-length *mTOR-001* based on BRCA-, CESC- and LIHC-TCGA datasets using GEPIA2. (**L**) Exon 8 skipping of *mTOR* was examined by 3% agarose gel electrophoresis of PCR products in *SF3B3*-knockdown MCF7, Hela and Huh7 cells transfected with siRNAs for 48 h. (**M**) Representative western blots of mTOR and LC3 in *SF3B3*-knockdown MCF7, Hela and Huh7 cells transfected with siRNAs for 72 h. Data are shown as mean ± SD.


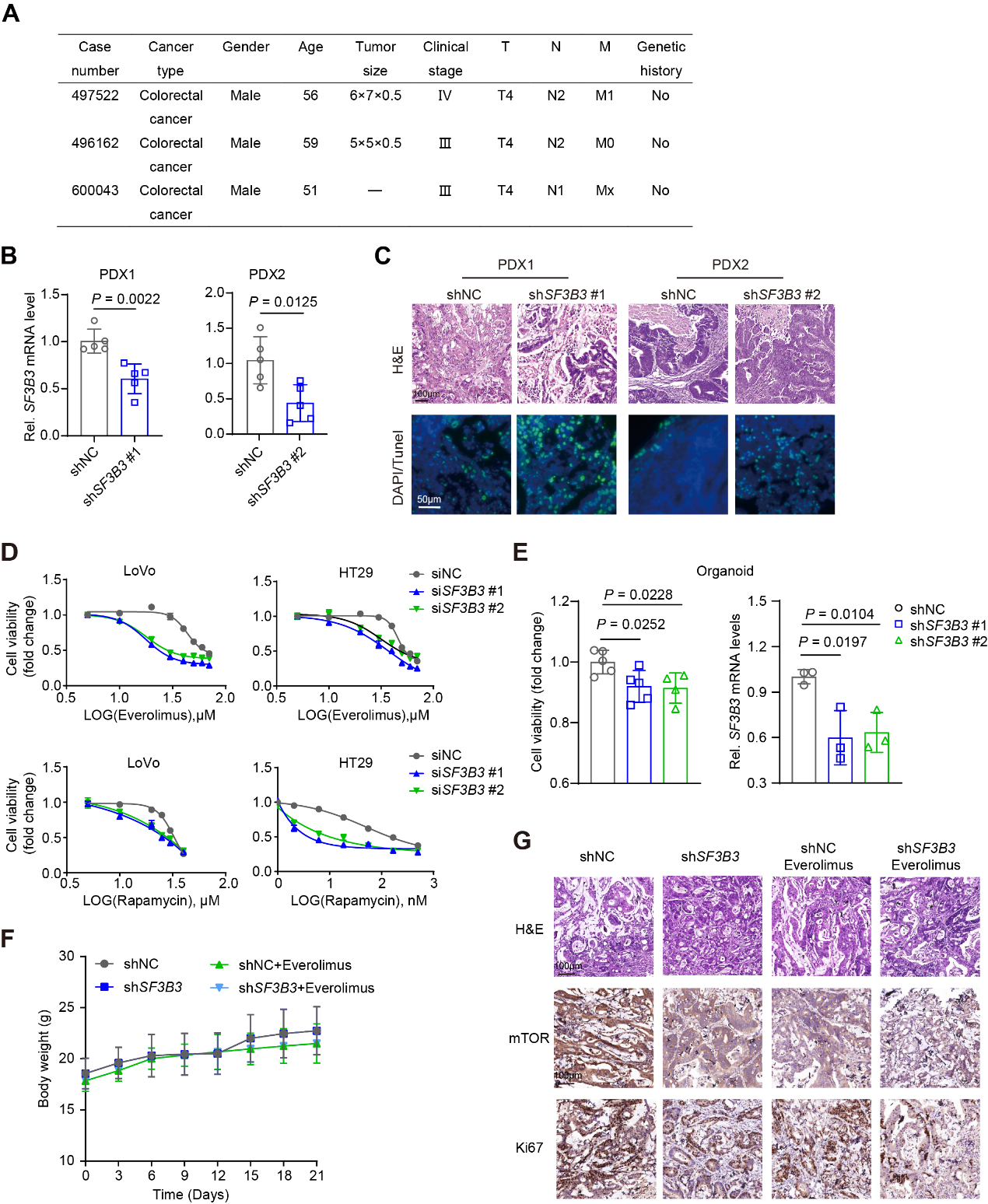


**Figure S7.** **Effect of *SF3B3* knockdown and mTOR inhibitor on CRC.** (**A**) The clinical information of three donor patients investigated in this study. (**B**) qRT-PCR verification of *SF3B3* knockdown in two PDX murine models. PDX1 models were intratumorally treated with sh*SF3B3*#1 lentivirus, whereas PDX2 models were treated with sh*SF3B3*#2 lentivirus. (**C**) Representative H&E staining and TUNEL staining (green) of two PDXs. Scale bars, 50 μm. (**D**) Cell viability of LoVo and HT29 cells after transfection with siRNAs for 24 h, followed by rapamycin or everolimus treatment at the indicated concentrations for 48 h. (**E**) Viability and qRT-PCR validation of *SF3B3-*knockdown CRC tumor organoids. Digested organoids were transduced with sh*SF3B3* lentivirus for 6 h, followed by reconstitution in matrigel for 7 days. (**F**) Body weights of PDX mice. Xenograft tumors were transplanted into left and right flanks of each nude mouse to establish PDXs. Mice were intratumorally injected with sh*NC* lentivirus on the left flank and sh*SF3B3* lentivirus (mixture of sh*SF3B3*#1 and sh*SF3B3*#2) on the right flank. After that, mice were orally administered vehicle (40%PEG400, 5% Tween-80 and 5% DMSO) or everolimus (5 mg/kg, every two days for 3 weeks). (**G**) Representative H&E staining as well as immunohistochemistry images of mTOR and Ki67 in PDX3 tumor tissues. Data are shown as mean ± SD.


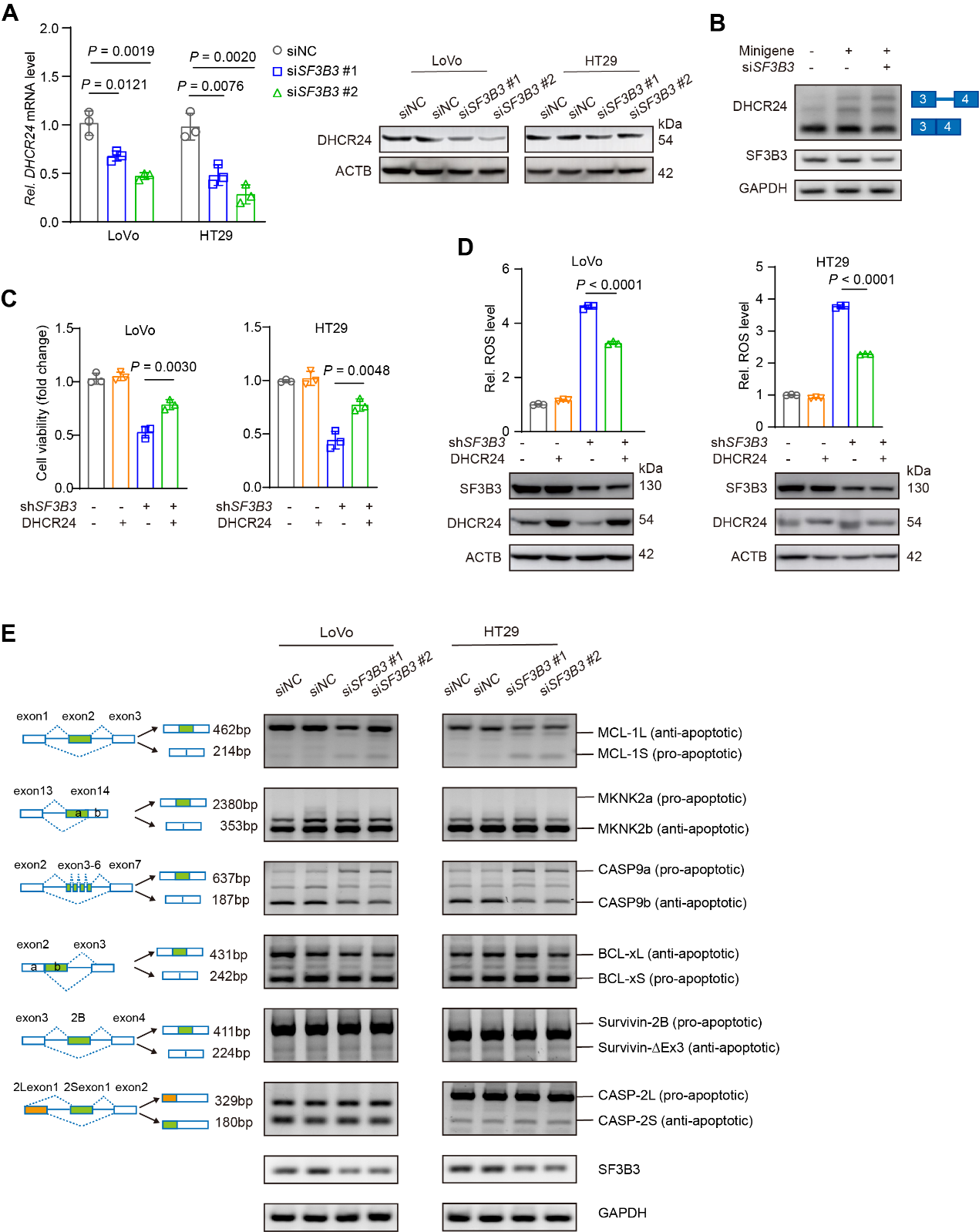


**Figure S8. SF3B3 regulates alternative splicing of *DHCR24*** **and apoptotic genes.** (**A**) mRNA and protein levels of DHCR24. CRC cells were transfected with siRNAs, and collected at 48 h for qRT-PCR and at 72 h for western blots. (**B**) Minigene detection showed the effects of *SF3B3* knockdown on *DHCR24* splicing in CRC cells. The minigene contains exons 3-4 of *DHCR24*. LoVo cells were transfected with *DHCR24* minigene together with *SF3B3* siRNAs for 48 h. (**C**) Detection of cell viability and (**D**) ROS in stably *SF3B3*-knockdown CRC cells transfected with DHCR24 overexpressing plasmid for 72 h. Data are shown as mean ± SD. (**E**) Alternative splicing of apoptotic genes was examined by 1-3% agarose gel electrophoresis of PCR products in CRC cells. LoVo and HT29 cells were transfected with siRNAs for 48 h.

**Supplemental Tables**

**Table S5.** Sequences of siRNAs.

| **Name** | **Forward** | **Reverse** |
| --- | --- | --- |
| siNC | UUCUCCGAACGUGUCACGUTT | ACGUGACACGUUCGGAGAATT |
| si*SF3B3* #1 | CCAGAUAUCCGCUGUCCAATT | UUGGACAGCGGAUAUCUGGTT |
| si*SF3B3* #2 | CCACGAAAGCUCAGAGAAATT | UUUCUCUGAGCUUUCGUGGTT |
| si*mTOR* | CCAAGGAGCUCCAGCACUATT | UAGUGCUGGAGCUCCUUGGTT |
| si*TSC1* | CGGCUGAUGUUGUUAAAUATT | UAUUUAACAACAUCAGCCGTT |
| si*TSC2* | GGGACAUUCUGCUGAACAUTT | AUGUUCAGCAGAAUGUCCCTT |

**Table S6.** Sequences for plasmid construction.

| **Name** | **Forward** | **Reverse** |
| --- | --- | --- |
| SF3B3-FL | TAAGCAGAATTCGCCACCATGTTTCTGTACAACTTAACC | TGCTTAGCGGCCGCTCAGAAGGCGTAGCGGGTCC |
| h-mTOR-mini | TAAGCAGAATTCATGGAAGAAATCACACAGCAGC | TGCTTAGCGGCCGCCGCGAGGCAAATAGACCTTAA |
| DHCR24-FL | TAAGCAGAATTCGCCACCATGGAGCCCGCCGTGTCGCTG | TGCTTAGCGGCCGCTCAGTGCCTGGCGGCCTTGCA |
| h-DHCR24-mini | TAAGCAGAATTCGACACCTTCTGTGTGCAGCA | TGCTTAGCGGCCGCACTGGGAATGAAGCACACCTA |
| h-pgl4-SREBF1a-promoter | TAAGCAGATATCAGGGAGCTCACTGCCTTATCA | TGCTTAAAGCTTCTCGGAAACTGGGTTCCCC |
| h-pgl4-SREBF1c-promoter | TAAGCAGATATCATCAACTGGCCAGGCTCTGA | TGCTTAAAGCTTTAGCGGACGTCCGCCTTTA |

**Table S7.** Sequences of shRNAs.

| **Name** | **Forward** | **Reverse** |
| --- | --- | --- |
| shNC | CCGGCCTAAGGTTAAGTCGCCCTCGCTCGAGCGAGGGCGACTTAACCTTAGGTTTTTG | AATTCAAAAACCTAAGGTTAAGTCGCCCTCGCTCGAGCGAGGGCGACTTAACCTTAGG |
| sh*SF3B3* #1 | CCGGGTTGGAGTAGATGTCGGATTTCTCGAGAAATCCGACATCTACTCCAACTTTTTG | AATTCAAAAAGTTGGAGTAGATGTCGGATTTCTCGAGAAATCCGACATCTACTCCAAC |
| sh*SF3B3* #2 | CCGGTGAGAGTAACAACCTTATTATCTCGAGATAATAAGGTTGTTACTCTCATTTTTG | AATTCAAAAATGAGAGTAACAACCTTATTATCTCGAGATAATAAGGTTGTTACTCTCA |

**Table S8.** Primer sequences for qRT-PCR or PCR.

| **gene** | **Forward primer sequence (5’-3’)** | **Forward primer sequence (5’-3’)** |
| --- | --- | --- |
| h*SF3B3* | GGCGAGCCGTTATGATTAGT | AGGTCTAGCTCATAGAAAGTAAG |
| h*DHCR24* | ATGGAAGGAGCAGGGTAGCA | AAGGGCTCCACACGGACAAT |
| h*FASN* | AACTCCAAGGACACAGTCACCAT | CAGCTGCTCCACGAACTCAA |
| h*SREBF1* | TGGTGCTCGTCTCCTTGGT | AGCCTGCTTGCGATGCCTC |
| h*SREBF1a* | GGAGCCATGGATTGCACTTTC | CCAGCATAGGGTGGGTCAAA |
| h*SREBF1c* | GCTGCTGACCGACATCGAA | GGGTGGGTCAAATAGGCCAG |
| h*SREBF2* | TTCTCCCACCTCAGTTCCCA | ACCAGGCTTTGGACTTGAGG |
| h*SREBF1a*-pcr | GCCAGGGCAGGACACGAA | CAATGTGGCAGGAGGTGGAGA |
| h*SREBF1c*-pcr | CGCCTTGACAGGTGAAGTCG | CAATGTGGCAGGAGGTGGAGA |
| h*ACTB* | CTCTTCCAGCCTTCCTTCCT | AGCACTGTGTTGGCGTACAG |
| h*ACACA* | TCAGTTTCCCAGCCAGCAGA | GCAGATCCATCACCACAGCCT |
| h*SCD* | ACCATTACAGCGCCTCCCT | ATCCCATACAGGGCTCCCA |
| h*ACLY* | TGAGTCCAGGGCCGATGA | CTTGGTGTGGCGGCTGAA |
| h*mTOR*-pcr | CTGGTGGAGAGCCGGTGTT | TGCCTTCTGCCTCTTATGGG |
| h*mTOR* | GTCTATTTGCCTCGCGTGC | GTGAGGGCAGGGCTTAGTC |
| *hmTOR*β-100bp | ATGCTTGGAACCGGACCTGCCG | TTTGGACAGATCCTCAGTGACCT |
| *hmTOR*β-2.1kb | ATGCTTGGAACCGGACCTGCCG | TACCAGAAAGGGCACCAGCCAAT |
| h*GAPDH* | GAAGGTCGGAGTCAACGGATT | CGCTCCTGGAAGATGGTGAT |
| h*EZH2* | AATCAGAGTACATGCGACTGAGA | GCTGTATCCTTCGCTGTTTCC |
| h*FOXM1* | TCCTTCTGGACCATTCACCC | ATTCCAAGTGCTCGGGCAA |
| MT-ND1 | CCTAATGCTTACCGAACGA | GGGTGATGGTAGATGTGGC |
| β-global | CTGCTGGTGGTCTACCCTTG | AGGCCATCACTAAAGGCACC |
| *SF3B3*-promoter | AAAGCAAAGCAATAAAGCATGT | AATCCGCGCCTGAAGAATAT |
| h*MCL1*-pcr | GCCAAGGACACAAAGCCAAT | TGCCAAACCAGCTCCTACTCC |
| h*MKNK2*-pcr | GCTGCGACCTGTGGAGCCTGGG | GAGGAGGAAGTGACTGTCCCAC |
| h*CASP9*-pcr | AAACACCCAGACCAGTGGACA | GGCAAACTAGATATGGCGTCC |
| h*BCL-x*-pcr | GGGAGGTGATCCCCATGGCAG | GGCCACAGTCATGCCCGTCAG |
| h*Survivin*-pcr | GCTTCATCCACTGCCCCAC | CAGCCACTCTGGGACCAGG |
| h*CASP2L*-pcr | TGTGCGTCCGCGTCTGAG | GGCCTGGATGAGCTCCCT |
| h*CASP2S*-pcr | AATGGAAGAAATCTGCTGCACC | GGCCTGGATGAGCTCCCT |

**Table S9.** List of antibodies used in this study

| **Antibody** | **Manufacturer** | **Catalog** | **Application** |
| --- | --- | --- | --- |
| SF3B3 | MilliporeSigma | HPA042986 | 1:2000 1:1000 for IHC |
| DHCR24 | Cell Signaling Technology | 2033 | 1:1000  1:100 for IHC |
| ACTB | BIOSS | bs-0061R | 1:5000 |
| mTOR | Cell Signaling Technology | 2983 | 1:1000  1:100 for IHC |
| mTOR | Santa Cruz Biotechnology | sc-517464 | 1:500 |
| P-mTOR (Ser2448) | Cell Signaling Technology | 2971 | 1:1000 |
| Cleaved Caspase-3 | Santa Cruz Biotechnology | sc-56053 | 1:500  1:100 for IHC |
| Cleaved Caspase-8 | Proteintech | 13423-1-AP | 1:1000 |
| Cleaved Caspase-9 | BOSTER | PB0285 | 1:1000 |
| cytochrome c | Santa Cruz Biotechnology | sc-13156 | 1:500 |
| Cleaved PARP | Cell Signaling Technology | 5625 | 1:1000 |
| LC3 | Cell Signaling Technology | 3868 | 1:1000  1:1600 for IF |
| Raptor | Cell Signaling Technology | 2280 | 1:1000 |
| Rictor | Cell Signaling Technology | 2114 | 1:1000 |
| p-4EBP1 (Ser65) | Cell Signaling Technology | 9451 | 1:1000 |
| SREBF1 | BBI | D264443 | 1:2000  1:200 for IHC |
| FASN | Cell Signaling Technology | 3180 | 1:1000  1:100 for IHC |
| ACACA | HUABIO | ET1609-77 | 1:1000 |
| ACLY | BBI | D221957 | 1:2000 |
| SCD | BBI | D262163 | 1:1000 |
| p62 | Cell Signaling Technology | 8025 | 1:1000 |
| p-S6K(Thr421/Ser424) | Cell Signaling Technology | 9204 | 1:1000 |
| Ki67 | absin | abs130135 | 1:100 for IHC |
| VIM | BBI | D120268 | 1:2500  1:100 for IHC |
| VIM | Santa Cruz Biotechnology | sc-373717 | 1:50 for IF |
| E-Cadherin | BBI | D160656 | 1:2500  1:100 for IHC |
| E-Cadherin | Santa Cruz Biotechnology | sc-8426 | 1:50 for IF |
| N-Cadherin | Cell signaling Technology | 13116 | 1:1000  1:50 for IHC |
| anti-rabbit IgG Alexa Fluor 488 | BOSTER | BA1127 | 1:500 |
| anti-rabbit IgG | Cell signaling Technology | 7074 | 1:5000 |
| anti-mouse IgG | Cell signaling Technology | 7076 | 1:5000 |
| H3K27ac | Abcam | ab4729 | 1:50 |
